# Recent Research Progress of Mn^4+^-Doped A_2_MF_6_ (A = Li, Na, K, Cs, or Rb; M = Si, Ti, Ge, or Sn) Red Phosphors Based on a Core–Shell Structure

**DOI:** 10.3390/nano13030599

**Published:** 2023-02-02

**Authors:** Yueping Xie, Tian Tian, Chengling Mao, Zhenyun Wang, Jingjia Shi, Li Yang, Cencen Wang

**Affiliations:** 1 School of Materials Science and Engineering, Shanghai Institute of Technology, Shanghai 201418, China; xie_yueping@163.com (Y.X.); mao_chengling@163.com (C.M.); 19861379051@163.com (Z.W.); gagamuse@163.com (J.S.); 2 Shanghai Toplite Technology Company Limited, Shanghai 201712, China; yangli_angel@foxmail.com (L.Y.); rain_cen@126.com (C.W.)

**Keywords:** Mn^4+^, A_2_MF_6_ fluoride, core–shell structure, red phosphor, water resistance

## Abstract

White light emitting diodes (WLEDs) are widely used due to their advantages of high efficiency, low electricity consumption, long service life, quick response time, environmental protection, and so on. The addition of red phosphor is beneficial to further improve the quality of WLEDs. The search for novel red phosphors has focused mainly on Eu^2+^ ion- and Mn^4+^ ion-doped compounds. Both of them have emissions in the red region, absorption in blue region, and similar quantum yields. Eu^2+^-doped phosphors possess a rather broad-band emission with a tail in the deep red spectral range, where the sensitivity of the human eye is significantly reduced, resulting in a decrease in luminous efficacy of WLEDs. Mn^4+^ ions provide a narrow emission band ~670 nm in oxide hosts, which is still almost unrecognizable to the human eye. Mn^4+^-doped fluoride phosphors have become one of the research hotspots in recent years due to their excellent fluorescent properties, thermal stability, and low cost. They possess broad absorption in the blue region, and a series of narrow red emission bands at around 630 nm, which are suitable to serve as red emitting components of WLEDs. However, the problem of easy hydrolysis in humid environments limits their application. Recent studies have shown that constructing a core–shell structure can effectively improve the water resistance of Mn^4+^-doped fluorides. This paper outlines the research progress of Mn^4+^-doped fluoride A_2_MF_6_ (A = Li, Na, K, Cs, or Rb; M = Si, Ti, Ge or Sn), which has been based on the core–shell structure in recent years. From the viewpoint of the core–shell structure, this paper mainly emphasizes the shell layer classification, synthesis methods, luminescent mechanism, the effect on luminescent properties, and water resistance, and it also gives some applications in terms of WLEDs. Moreover, it proposes challenges and developments in the future.

## 1. Introduction

Since the advent of the white light emitting diode (WLED), it has been studied and applied in backlight displays, lighting devices, fast imaging, and other aspects based on its advantages of high efficiency, environmental protection, durability, low energy, and so on [1,2,3,4,5,6,7,8]. The combination of a blue InGaN chip and yellow Y_3_Al_5_O_12_: Ce^3+^ (YAG: Ce^3+^) phosphors was applied to the first commercial phosphor-converted white LED (pc-WLED) to produce white light and achieve a wide range of commercial applications [9,10,11]. It is known that adding red phosphors is beneficial to the white light emission of this WLED with high CRI and low CCT [12,13]. A great deal of research has focused on discovering new types of red phosphors. The search for novel red phosphors has been mainly focused on two alternative activator ions, namely, Eu^2+^ and Mn^4+^, doped in various hosts [14]. The overall characteristics of the two activators are very similar; Eu^2+^ possesses emission peaks in the range of 600–650 nm, and Mn^4+^ produces emission peaks at 630 nm. In addition, the lowest energy excitation peak of Eu^2+^ ions is located at ~475 nm, and the long wavelength absorption edge extends to ~650 nm, while the same eigenvalues of Mn^4+^ ions are ~450 and ~500 nm, respectively. However, the emission spectra of Eu^2+^-doped phosphors (such as CaAlSiN_3_: Eu^2+^ and Sr_2_Si_5_N_8_: Eu^2+^) are broad-band, most of which cover wavelengths longer than 650 nm, which limits the maximum luminous efficiency of WLEDs because the human eye is insensitive to deep red light with wavelengths longer than 650 nm [15]. At the same time, the synthesis conditions of some Eu^2+^-doped phosphors are quite harsh, which cause the production cost to be high [16]. These shortcomings limit the application of Eu^2+^ in efficient WLEDs. Red phosphors with emission peaks in the wavelength range of 590–650 nm and a cutoff absorption edge shorter than 510 nm are conductive to improving the performance of WLEDs. Obviously, Mn^4+^ ions with ^3^d_3_ electronic structures are suitable candidates. Manganese ion (Mn^4+^) exhibits broad absorption in the blue light region and narrow emission lines at ~630 nm in fluoride hosts and ~670 nm in oxide hosts because of its distinctive electronic structure [17,18,19]. The broad emission peaks of Mn^4+^-doped oxides exceed 650 nm, which is almost unrecognizable by the human eye and causes high energy loss in WLED [20]. In contrast, fluoride is an ideal luminescent matrix due to its high thermal stability, structural diversity, and low phonon energy. Mn^4+^-doped fluoride red luminescent materials exhibit narrow-band linear luminescence, high thermal stability, and high quantum efficiency, which have attracted wide attention among researchers.

Mn^4+^-doped fluoride phosphors were first reported in 1973 and have expanded from A_2_MF_6_: Mn^4+^ to AMF_5_: Mn^4+^, A_3_MF_6_: Mn^4+^, A_2_A′MF_6_: Mn^4+^, and A_3_MF_7_: Mn^4+^ [21,22,23,24,25,26,27]. Among them, A_2_MF_6_ (A = Li, Na, K, Cs, or Rb; M = Si, Ti, Ge, or Sn): Mn^4+^ red phosphor is homovalently doped and has a similar radius of Mn^4+^ ions and M^4+^ ions, which makes synthesis easier. Moreover, it has been targeted for particular focus due to its high purity and excellent spectral property [28,29,30]. However, the most severe disadvantage of Mn^4+^-doped fluoride is its poor chemical stability or water resistance. It was discovered that Mn^4+^-doped fluoride was very sensitive to humidity, and the Mn^4+^-dopant on the surface effortlessly hydrolyzes into manganese oxide and hydroxide with mixed valence, which can cause the color of the phosphor to be darkish and reduce the emission intensity [31].

**Figure 1:**
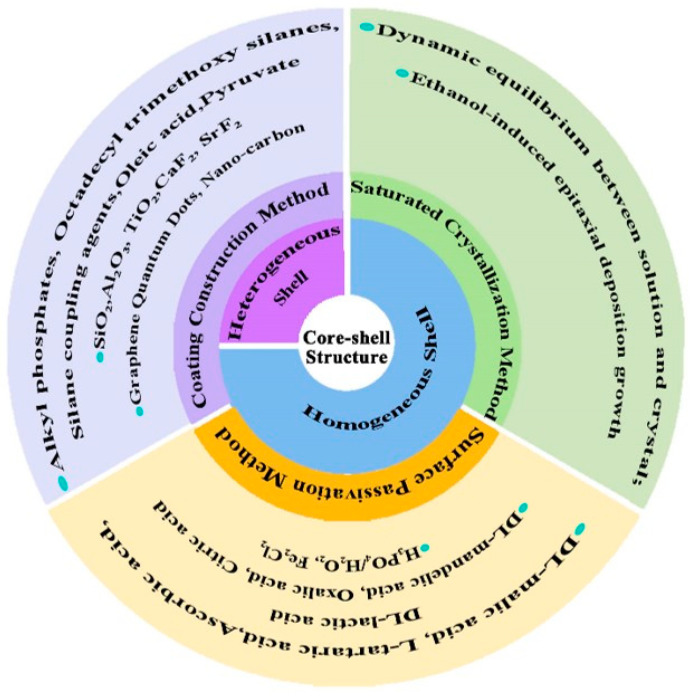
The outline of the classification and synthesis methods of a core–shell structure for A_2_MF_6_: Mn^4+^.

Phosphor is an important component of WLED, and its water resistance is closely related to the life of the device. Therefore, improving the water resistance of fluoride is of great importance for its application in WLED. A core–shell structure is an ordered assembly structure shaped by using one material coating another material through chemical bonds or different forces. Its distinctive structural features combine the advantages of the two substances and complement each other’s shortcomings, offering a way to increase the water resistance of Mn^4+^-doped fluoride. Reviews of the structure, green synthesis route, and thermal properties of Mn^4+^-doped fluorides have been reported [18,32,33,34]. Up to now, to the best of our knowledge, reviews of Mn^4+^-doped A_2_MF_6_ (A = Li, Na, K, Cs, or Rb; M = Si, Ti, Ge, or Sn) red phosphors based on a core–shell structure have not been reported. This review mainly introduces the recent research progress of Mn^4+^-doped A_2_MF_6_ (A = Li, Na, K, Cs, or Rb; M = Si, Ti, Ge, or Sn) red phosphors with a core–shell structure. Especially from the viewpoint of a core–shell structure, as shown in Figure 1, it mainly summarizes the layer classification, synthetic methods, luminescent mechanism, the effect on luminescent properties, and water resistance of Mn^4+^-doped A_2_MF_6_. In addition, some of their applications in WLED are also given. Furthermore, prospective challenges and developments in the future are also discussed.

## 2. Classification of the Shell Layer in A_2_MF_6_: Mn^4+^

In practice, [MnF_6_]^2−^ in Mn^4+^-doped fluoride is easily hydrolyzed to brown MnO_2_, which deteriorates the luminescence and generates HF, leading to the packaging corrosion and failure of WLED. Therefore, it is of extraordinary value to enhance the water resistance of fluorides. In general, coating the surface of A_2_MF_6_: Mn^4+^ with hydrophobic materials to form a core–shell structure can improve their water resistance. The shell layer can be divided into heterogeneous and homogeneous shell layers.

### 2.1. Heterogeneous Shell Layer

The heterogeneous shell layer means that the shell material is different from the matrix material of the core. It connected with the core through chemical bonds or other interactions. Common heterogeneous shell layer materials are alkyl phosphates, octadecyl trimethoxy silanes (ODTMS), silane coupling agents, oleic acid (OA), CaF_2_, SrF_2_, Al_2_O_3_, TiO_2_, SiO_2_, GQDs (graphene quantum dots), and nano-carbon, which can act as water repellents to improve the chemical stability or the water resistance of A_2_MF_6_: Mn^4+^.

Nguyen et al. [35] successfully coated the K_2_SiF_6_: Mn^4+^ surface with an alkyl phosphate layer to enhance its chemical stability. Zhou et al. [36] used ODTMS to significantly improve the moisture resistance and thermal stability of K_2_TiF_6_: Mn^4+^. Kim et al. [37] modified the surface of K_2_SiF_6_: Mn^4+^ with silane coupling agents. The formation of a hydrophobic shell increased its water resistance, and the removal of surface quench sites enhanced its emission efficiency. The results of these studies suggested that the surface modification of hydrophobic silane coupling agent was an effective method to improve the humidity of fluoride phosphor, which has practical application potential. Arunkumar et al. [38] modified the K_2_SiF_6_: Mn^4+^ surface with OA, and Fang et al. [39] used SiO_2_ and OA to form a double-coated KTF@OA@SiO_2_ phosphors. Both improved the water resistance of K_2_SiF_6_: Mn^4+^. In addition, Luo et al. [40] treated K_2_SiF_6_: Mn^4+^ (KSFM) with pyruvate in one step to construct an impermeable dual-shell-stabilized fluoride phosphor, namely, KSFM-98PA.

CaF_2_ has good chemical stability and is also suitable for improving the chemical stability of A_2_MF_6_: Mn^4+^. Dong et al. [41] and Yu et al. [42] modified the surface of K_2_TiF_6_: Mn^4+^ and K_2_SiF_6_: Mn^4+^ with CaF_2_, which strengthened the humidity resistance and luminescence properties. Fang et al. [43] constructed a water-resistant SrF_2_ coating on the surface of K_2_TiF_6_: Mn^4+^. SrF_2_ was uniformly covered on K_2_TiF_6_: Mn^4+^ to eliminate lattice defects and improve the emission efficiency. Verstraete et al. [44] deposited a TiO_2_ or Al_2_O_3_ layer on the surface of K_2_SiF_6_: Mn^4+^ and obtained K_2_SiF_6_: Mn^4+^-TiO_2_ and K_2_SiF_6_: Mn^4+^-Al_2_O_3_, respectively. Kate et al. [45] deposited ultrathin Al_2_O_3_ on the surface of K_2_SiF_6_: Mn^4+^ with trimethyl aluminum (TMA) to improve its optical properties, chemical stability, and thermal stability. Quan et al. [46] modified a layer of SiO_2_ on the surface of K_2_SiF_6_: Mn^4+^ to further improve the water resistance of the material.

In addition, carbon materials can also be applied to improve the water resistance of A_2_MF_6_: Mn^4+^. GQDs have the characteristics of large specific surface area, conjugated large π bonds, multiple uses, environmental friendliness, and good thermal stability. Yu et al. [47] constructed a double shell structure on the surface of K_2_SiF_6_: Mn^4+^, Na^+^ to form K_2_SiF_6_: Mn^4+^, Na^+^@GQDS@K_2_SiF_6_ phosphors with water-resistance. Liu et al. [48] modified carbon nanoparticles on the surface of K_2_SiF_6_: Mn^4+^, and C atoms combined with F atoms in K_2_SiF_6_: Mn^4+^ to form carbon–fluorine (C-F) covalent bonds. Carbon has low polarizability and excellent hydrophobicity, which are beneficial for improving the water resistance of K_2_SiF_6_: Mn^4+^.

**Figure 2:**
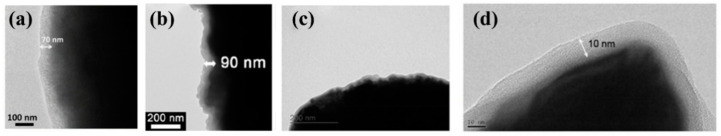
TEM images of K_2_SiF_6_/K_2_TiF_6_ with heterogeneous core–shell structures. (**a**) K_2_SiF_6_@MOPAl (Reprinted from Ref [35]. Copyright from John Wiley and Sons Ltd., New York, NY, USA, 2015); (**b**) K_2_TiF_6_@ODTMS (Reprinted from Ref [36]. Copyright from American Chemical Society, 2018); (**c**) K_2_SiF_6_@OA (Reprinted from Ref [38]. Copyright from American Chemical Society, 2017); (**d**) K_2_TiF_6_@OA@SiO_2_ (Reprinted from Ref [39]. Copyright from American Chemical Society, 2018).

As summarized above, the matrix materials involved in the construction of heterogeneous core–shell structures are mainly K_2_SiF_6_ and K_2_TiF_6_, probably due to their excellent optical properties, good luminescence efficiency, and high quantum efficiency, which can effectively reduce the luminescence loss caused by the shell. When the phosphor was coated with organic material, a film layer on the surface could be clearly seen by transmission electron microscopy (TEM) at different magnifications. Figure 2 shows the TEM images of typical organic coating layers on the surface of K_2_SiF_6_/K_2_TiF_6_ with a heterogeneous core–shell structure. The surface shell layer of K_2_SiF_6_@MOPAl and K_2_SiF_6_@OA were affected under the electron beam of a TEM system, leading to decomposition. Moreover, the desorption on the surface leads to the formation of KF and other corrosive substances. They corrode the core–shell interface and destroy the good adhesion between the core and shell, which may result in fluorescence quenching.

### 2.2. Homogeneous Shell Layer

A homogeneous shell layer can be defined as the material of the shell that is the same as the material of the core. Because the material of the core and shell are the same, and due to the high matching degree between them, the fluorescence performance may be enhanced. The homogeneous core–shell structure of A_2_MF_6_: Mn^4+^ can be constructed by different methods, removing the Mn^4+^ ions on surface, and leaving the layer matrix material that is the same as the core.

Huang et al. [49] successfully obtained KGFM@MA by loading DL-mandelic (MA) on the surface of K_2_GeF_6_: Mn^4+^(KGFM) to improve water resistance. They also used H_3_PO_4_ and H_2_O_2_ aqueous solutions to promote the release and decomposition of [MnF_6_]^2−^ ions on the K_2_SiF_6_: Mn^4+^(KSFM) surface and converted the KSFM surface into KSF, finally forming a uniform KSFM@KSF composite structure on the surface to improve water resistance [50]. Zhou et al. [51] made passivation of K_2_XF_6_: Mn^4+^(KXF, X = Ti, Si, Ge) with H_2_O_2_, H_2_O_2_, and [MnF_6_]^2−^ through a chemical reaction in an acidic environment, which reduced the distribution of Mn^4+^ on the surface of K_2_XF_6_: Mn^4+^ and collected phosphors, and the surface redox treatment by H_2_O_2_ (P-KXF) with good water resistance. Liu et al. [52] passivated Cs_2_SiF_6_: Mn^4+^ (CSFM) with H_2_O_2_ and collected surface passivation phosphors (p-CSFM) with good water resistance. Yu et al. [53], Jiang et al. [54], and Liu et al. [55] used an oxalic acid solution, and Li et al. [56] and Zhong et al. [57] used a citric acid or oxalic acid solution. Moreover, Cai et al. [58] utilized Fe^2+^ to passivate [MnF_6_]^2−^ on the surface of phosphor to constructed a uniform Mn^4+^-free surface layer to improve the water resistance of phosphor. The shell materials of T-K_2_GeF_6_, Rb_2_SnF_6_: Mn^4+^, R-K_2_SiF_6_: Mn^4+^, P1-CsNaGe_0.5_Sn_0.5_F_6_, LiNaSiF_6_: Mn^4+^-CA, and K_2_SiF_6_-T series phosphors with homogeneous low solubility and Mn^4+^-free surfaces were obtained, respectively.

**Figure 3:**
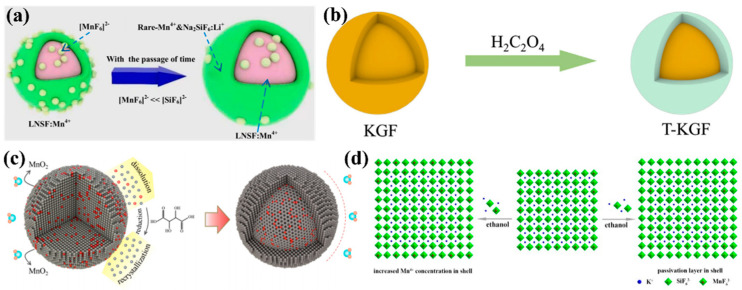
The model diagram of homogeneous core–shell structure formation. (**a**) LiNaSiF_6_: Mn^4+^ (Reprinted from Ref [60]. Copyright from American Chemical Society, 2022); (**b**) T-KGF (Reprinted from Ref [53]. Copyright from Elsevier, Amsterdam, The Netherlands, 2018); (**c**) KSFM-RSRC (Reprinted from Ref [29]. Copyright from Elsevier, 2021); (**d**) K_2_SiF_6_: Mn^4+^ (Reprinted from Ref [61]. Copyright from Royal Society of Chemistry, 2021).

Wan et al. [29] obtained KSFM-RSRC with good water resistance by using reduction-assisted surface recrystallization (RSRC) to treat K_2_SiF_6_: Mn^4+^, remove surface Mn^4+^ ions, and construct their own encapsulated shell structure. Li et al. [59] synthesized K_2_SiF_6_: Mn^4+^@K_2_SiF_6_ with good water resistance, luminescent thermal stability, and high efficiency by the coating K_2_SiF_6_ on the core surface. Huang et al. [28] constructed K_2_TiF_6_: Mn^4+^@K_2_TiF_6_ with a self-coating structure. The outer shell not only prevented the hydrolysis of internal [MnF_6_]^2−^ groups in the air but also effectively blocked the path of energy transfer to surface flaws and further increased emission efficiency. Zhou et al. [30] and Zhong et al. [60] synthesized K_2_SiF_6_: Mn^4+^ and LiNaSiF_6_: Mn^4+^ with almost no Mn^4+^ surfaces by gradually reducing [MnF_6_]^2−^ on the crystal surface over time based on the dynamic equilibrium between dissolution and crystallization. Jiang et al. [61] synthesized K_2_SiF_6_: Mn^4+^@K_2_SiF_6_ by the ethanol-induced epitaxial deposition growth of fluoride. These constructed homogeneous shells were effective in enhancing the water resistance of the cores. Figure 3 shows the model diagram of homogeneous core–shell structure formation. Mn^4+^ ions are mainly distributed in the core and on the surface with the Mn^4+^-free layer. The matrix materials for the construction of homogeneous core–shell structure are more diverse, including K_2_SiF_6_, K_2_TiF_6_, K_2_GeF_6_, and cationic iso-alkali double-doped matrix materials, which enrich the types of homogeneous shell matrix materials. The homogenous shell generated in situ can be uniformly wrapped on the surface of the core, avoiding lattice mismatch and further improving the emission efficiency of fluoride.

## 3. Preparation Methods of Shell Layer in A_2_MF_6_: Mn^4+^

There are usually two strategies to construct the core–shell structure. One is to nucleate first and then construct the shell, while the other is to form a core–shell structure at one time. For A_2_MF_6_: Mn^4+^, the former is suitable for the synthesis of both heterogeneous shells and homogeneous shells, while the latter is mainly suitable for the synthesis of homogeneous shells. There are mainly three preparation methods including the coating construction method, the surface passivation method, and the saturated crystallization method. The materials that making up the shell structure are different, so the properties of the fluoride also change [62].

### 3.1. Coating Construction Method

**Figure 4:**
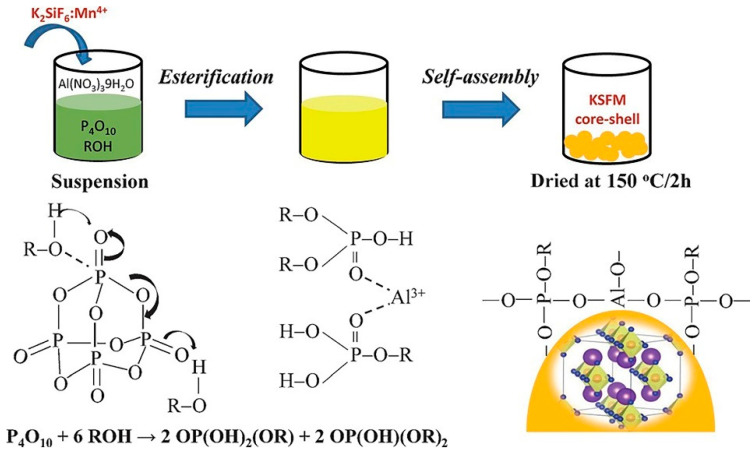
Formation of the alkyl phosphate layer on the K_2_SiF_6_: Mn^4+^ surface. (Reprinted with permission from Ref [35]. Published by Angewandte Chemie, 2015).

Nguyen et al. [35] prepared red K_2_SiF_6_: Mn^4+^ (KSFM) phosphors by the two-step co-precipitation method and added KSFM to the solution of Al(NO_3_)_3_, P_2_O_5_, and CH_3_OH (M). Figure 4 shows the synthesis diagram of KSFM-MOPAl. Al^3+^ ions act as the cross-linking agent between alkyl groups, and the P-O bond is broken and partially replaced by alkyl groups, forming a M-O-P-O-M bond and generating an organophosphorus layer. Through the adsorption mechanism, a network is formed on the surface of KSFM, and KSFM-MOPAl is obtained. Zhou et al. [36] added K_2_TiF_6_: Mn^4+^ to octadecyl trimethoxysilane and collected K_2_TiF_6_@ODTMS after stirring vigorously for 2 h. Kim et al. [37] introduced different silane materials via mixing with steam of isopropyl alcohol (IPA) and ammonium hydroxide (NH_4_OH) into the plasma reactor, respectively. After 20 min of plasma treatment, modified K_2_SiF_6_: Mn^4+^(KSFM) was obtained. Arunkumar et al. [38] dissolved oleic acid (OA) in absolute ethanol, added K_2_SiF_6_: Mn^4+^, dispersed it in the above solution for 1 h, then heated the mixed solution in a reaction kettle at 140 °C for 6 h. Finally, KSF-OA with a shell structure was formed. Fang et al. [37] used HF to remove impurities and small particles on the surface of K_2_TiF_6_: Mn^4+^(KTF) by the surface etching method, so that the surface of KTF was smooth. OA and SiO_2_ coatings were constructed by the hydrothermal method and the stirring method at room temperature to obtain a double-coated structural material, namely, KTF@OA@SiO_2_. Luo et al. [40] added K_2_SiF_6_: Mn^4+^ to pyruvate (PA, 98%), stirred it for 6 h, washed it with ethanol, and dried it to obtain KSRM-98PA. PA first reacted with Mn^4+^ on the surface to construct a Mn^4+^-free layer on the surface. In addition to reduction, PA could also form a soft shell on the fluoride surface through chemical bonds, which could prevent Mn^4+^ from reacting with water molecules on the surface.

Dong et al. [41] added K_2_TiF_6_: Mn^4+^ to a KF and Ca(NO_3_)_2_ solution, and Yu et al. [63] added K_2_SiF_6_: Mn^4+^ to a HF and Ca(NO_3_)_2_ solution via mixing and stirring, resulting in the obtaining of KTF@CaF_2_ and KSF: Mn^4+^ @CaF_2_, respectively. Fang et al. [43] used K_2_TiF_6_: Mn^4+^, KHF_2_, and Sr(NO_3_)_2_ as raw materials to obtain K_2_TiF_6_: Mn^4+^@SrF_2_. KHF_2_ played a bridging role in the synthesis of the shell structure, which could alleviate lattice mismatch. When K_2_TiF_6_: Mn^4+^(hexagonal phase) powder was immersed in a KHF_2_(cubic phase) solution, the free [HF_2_]^−^ could easily replace F^−^ ions in K_2_TiF_6_ and form a KHF_2_ thin layer on the surface of K_2_TiF_6_. After adding Sr^2+^ ions, KHF_2_ provided F^−^ ions for the nucleation growth of SrF_2_ particles on the KHF_2_ surface. Based on the chemical precipitation reaction of heterogeneous nucleation, the modification formed a denser and homogeneous coating on the surface of K_2_TiF_6_: Mn^4+^. Verstraete et al. [44] used atomic layer deposition (ALD) of TiO_2_ or Al_2_O_3_ to form a hydroxyl terminated functional seed layer on the K_2_SiF_6_: Mn^4+^ surface. The functionalized particle surface provided enhanced adhesion properties with conventional hydrophobic or moisture-resistant shells compared to the fluorine-terminated surface of the untreated phosphor. K_2_SiF_6_:Mn^4+^-Al_2_O_3_ and K_2_SiF_6_:Mn^4+^-TiO_2_ core–shell phosphors were obtained by this method. Kate et al. [45] used the vapor deposition method to deposit ultra-thin Al_2_O_3_ on the surface of K_2_SiF_6_: Mn^4+^ with trimethyl aluminum, O_3_, and N_2_ as raw materials. Quan et al. [46] synthesized K_2_SiF_6_: Mn^4+^@SiO_2_ (KSF@SiO_2_) by using tetraethyl orthosilicate (TEOS), isopropanol, H_2_O_2_, K_2_MnO_4_, KF·2H_2_O, sodium dodecyl benzene sulfonate (SDBS), and HF as raw materials. Li et al. [59] used KF·2H_2_O, KMnO_4_, HF (40%), and K_2_SiF_6_ as raw materials, and the nuclear material K_2_SiF_6_: Mn^4+^ was added to the HF solution of K_2_SiF_6_ and stirred for 2 h to obtain the final product K_2_SiF_6_: Mn^4+^@SiO_2_ (KSF@SiO_2_).

Yu et al. [47] mixed K_2_SiF_6_: Mn^4+^, Na^+^, GQD, and HF solution and transferred them to the reactor. K_2_SiF_6_: Mn^4+^ and Na^+^@GQDs were obtained by holding at 120 °C for 3 h. the K_2_SiF_6_: Mn^4+^, Na^+^@GQDs@KSF double shell layer structure was finally achieved by stirring K_2_SiF_6_ in HF solution. On the basis of improving water resistance, the GQDs coating material not only improved the luminescence intensity of the phosphor, but it also has a negative thermal quenching effect (NTQ) at higher temperatures. Liu et al. [48] synthesized K_2_SiF_6_: Mn^4+^@C by using chemical vapor deposition to decompose acetylene at a high temperature to generate a nano-carbon layer and form a hydrophobic protective layer on the surface of phosphor.

The experimental results showed that the coating construction method can successfully assemble the core–shell structure and enhance the water resistance of the fluoride. However, its process is complex, and there are some special requirements on the reaction conditions and the equipment operated in the experiment. The experiments may be carried out under high temperature and pressure conditions, while even needed to use toxic and volatile HF, which increases the safety hazards. Moreover, the equipment used to deposit the shell is usually expensive. Though constructing a double-layer coating could further enhance the water resisting property, the experimental steps are more complicated, and the difficulty of the experimental is also increased.

### 3.2. Surface Passivation Method

The surface passivation method is another strategy to improve the water resistance of fluoride. A homogeneous shell usually adopts the surface passivation method, in which Mn^4+^ ions are removed from the surface by a reducing agent, leaving the matrix material in situ to form a protective shell. This method prevents the formation of crystal defects at the core–shell interface caused by non-uniform coating and reduces fluorescence quantum yields (PLQYs).

**Figure 5:**
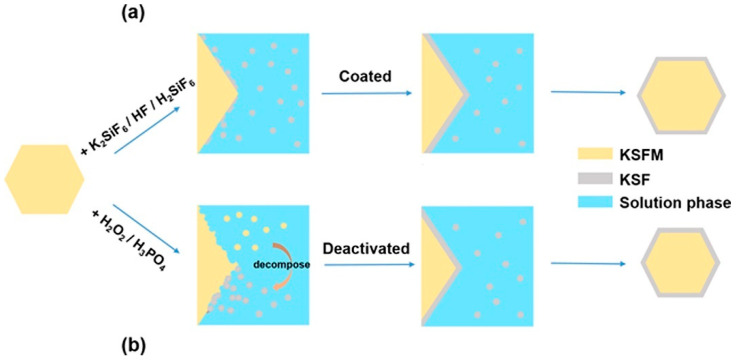
Design method of KSFM@KSF composites: (**a**) coated and (**b**) deactivated (Reprinted from Ref [50]. Copyright from American Chemical Society, 2018).

Huang et al. [49] synthesized K_2_GeF_6_:Mn^4+^ (KGFM) red phosphors by the three-step chemical coprecipitation method. KGFM was dissolved in the mixed solution of DL-mandelic acid and ethanol, and it was stirred for 2 h to obtain the final product KGFM@MA. Huang et al. [50] also synthesized K_2_SiF_6_: Mn^4+^@K_2_SiF_6_ by the coating and surface passivation methods, respectively, as shown in Figure 5a,b. Among them, the surface passivation method was use to dissolve K_2_SiF_6_: Mn^4+^ in the mixed aqueous solution of H_3_PO_4_/H_2_O_2_, which promoted the release and decomposition of [MnF_6_]^2−^ on the surface, so that the K_2_SiF_6_:Mn^4+^ surface was converted into K_2_SiF_6_ and the product WR-KFSM-8 was obtained. Zhou et al. [51] added K_2_XF_6_: Mn^4+^(KXF, X = Ti, Si, Ge) to H_2_O_2_ (30 wt%) solution and stirred it to reduce the distribution of Mn^4+^ ions on the surface of K_2_XF_6_: Mn^4+^. After washing with acetic acid and ethanol, the final product (P-KXF) was collected. The luminescence properties of hydrolyzed fluorine phosphors can be repaired by adding H_2_O_2_. Liu et al. [52] synthesized Cs_2_SiF_6_: Mn^4+^ phosphor (P-CSFM) by the same method. [MnF_6_]^2−^ existed on the surface of P-CSFM phosphor and could be effectively reduced by reacting with H_2_O_2_ in an acidic environment.

Yu et al. [53] added K_2_GeF_6_: Mn^4+^ into oxalic acid solution and stirred for 12 h. The interface between K_2_GeF_6_: Mn^4+^ and the solution was ionized, releasing K^+^ ions, [GeF_6_]^2−^, and [MnF_6_]^2−^, where [MnF_6_]^2−^ was reduced to Mn^2+^. The free [GeF_6_]^2−^ anion was combined with K^+^ ions to form K_2_GeF_6_, which was deposited on the surface of K_2_GeF_6_: Mn^4+^ to form K_2_GeF_6_: Mn^4+^@K_2_GeF_6_(T-KGF). Jiang et al. [54] added Rb_2_SnF_6_: Mn^4+^ to the oxalic acid solution, and they stirred and passivated Rb_2_SnF_6_: Mn^4+^ with oxalic acid to construct a passivation protective layer on the surface of. Liu et al. [55] employed the “good from bad” method to prepare R-KSFM by hydrolyzing the commercial K_2_SiF_6_: Mn^4+^ (O-KSFM) phosphors in deionized water for 10 min and then poured the oxalic acid solution into the above solution and stirred for 1 h. The collected R-KSFM not only fully recovered the luminescence properties but also possessed high moisture resistance. Li et al. [56] synthesized bicentric ions CsNaGe_0.5_Sn_0.5_F_6_: Mn^4+^(CNGSF) phosphors by the ethanol crystallization method. CNGSF was added into the weak reducing agent solution composed of citric acid or oxalic acid, and then stirring was carried out for the production of P1-CNGSF and P2-CNGSF samples. Zhong et al. [57] added the prepared Na_2_SiF_6_: Mn^4+^, Li^+^ (LNSF: Mn^4+^) into the KF-HF solution and stirred it; then they added aqueous citric acid solution and maintained stirring for 12 h to obtain the product of LNSF: Mn^4+^ with a shell layer (LNSF: Mn^4+^-CA). A protective layer with low solubility was in situ-formed on the surface, which could effectively isolate Mn^4+^ from the aqueous layer and help to improve the waterproof performance of LNSF: Mn^4+^-CA. In order to achieve the in situ formation of K_2_SiF_6_ on the surface of the K_2_SiF_6_: Mn^4+^ red phosphor particle, Cai et al. [58] put K_2_SiF_6_: Mn^4+^ phosphor into FeCl_2_ solution and utilized Fe^2+^ as a reducing agent to produce T-KSF phosphor with excellent water resistance. Wan et al. [29] proposed the new idea of reconstructing Mn^4+^-free shells of fluoride by reduction-assisted surface recrystallization (RSRC). The synthesized K_2_SiF_6_: Mn^4+^ was added to a reducing agent containing α-hydroxyl groups, such as L-tartaric acid (TA), DL-malic acid (MA), citric acid (CA), ascorbic acid (AA), or DL-lactic acid solution (LA), forming saturated K_2_SiF_6_ solution and stirring for 2 h to obtain serial phosphors. During the dissolution–crystallization equilibrium process of fluoride crystallization in HF, the reducing agent removed Mn^4+^ ions from the solution to prevent Mn^4+^ from re-entering the surface of the fluoride crystals to construct a Mn^4+^-free shell. Specifically, Huang et al. [28] constructed K_2_TiF_6_: Mn^4+^@K_2_TiF_6_ phosphors with a uniform coating core–shell structure based on the reverse cation exchange reaction. K_2_TiF_6_: Mn^4+^ microcrystals were synthesized by replacing Ti^4+^ ions in K_2_TiF_6_ crystals with Mn^4+^ ions in HF solution. Notably, the cation exchange process was reversible in nanocrystals. This reverse process was also applicable to K_2_TiF_6_: Mn^4+^ crystals. Using K_2_TiF_6_ as raw material, Ti^4+^ ions replaced Mn^4+^ of K_2_TiF_6_: Mn^4+^ in HF solution, leaving the K_2_TiF_6_ shell to protect the internal K_2_TiF_6_: Mn^4+^ core from ambient moisture and to prevent hydrolysis of [MnF_6_]^2−^.

The surface passivation method can complete the construction of the shell at room temperature, which is an effective method to construct the core–shell structure because of its simple operation and easily available raw materials. By using an acidic substance containing an α-hydroxy group as a reducing agent, the content of Mn^4+^ ions on the surface of the material can be appropriately decreased, and a protective layer can be formed without reducing the original luminescence. The protective layer can enhance the water resistance of A_2_MF_6_: Mn^4+^ by preventing the hydrolysis of the nuclear luminescence center.

### 3.3. Saturated Crystallization Method

In general, the crystallization process of crystals includes nucleation and crystal growth. The crystallization process of A_2_MF_6_: Mn^4+^ does not need to add additional heterogeneous materials and reducing agents. The saturated solution will precipitate crystals with the passage of time. During this process, some Mn^4+^ ions will generate Mn compounds with other valence states, making their concentrations change. The concentration difference between [MnF_6_]^2−^ and matrix ions can precipitate crystals with fewer Mn^4+^ ions on the surface. What is more, the single crystal phosphor has the advantages of high crystallinity, few defects, and high thermal conductivity.

**Figure 6:**
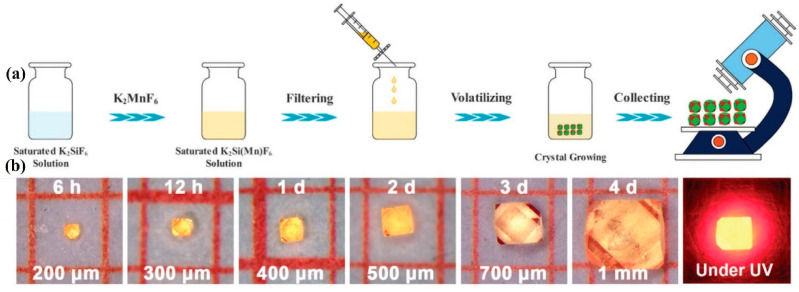
(**a**) Schematic diagram of the preparation process of KSFM single crystal; (**b**) pictures of KSFM single crystal under visible light and UV light collected at different times. (Reprinted from Ref [30]. Copyright from American Chemical Society, 2018).

Zhou et al. [30] mixed H_2_SiF_6_, KF, K_2_SiF_6_, HF, and K_2_MnF_6_ and stirred vigorously to form K_2_SiF_6_/K_2_MnF_6_ saturated solution. They filtered the mixed solution with a filter and placed it in a fume hood. After volatilizing it at room temperature for a period of time, K_2_SiF_6_: Mn^4+^(KSFM) crystals could be grown from the saturated solution, as shown in Figure 6a. During the process of crystal growth, [SiF_6_]^2−^ groups did not hydrolyze in acidic solution, while its concentration decreased with the precipitation of KSFM single crystals. However, [MnF_6_]^2−^ were prone to disproportionation reactions to produce other compounds (Mn^2+^, [MnO_4_]^−^) of manganese. With the growth time prolonging, the concentration of [MnF_6_]^2−^ decreased more rapidly than that of [SiF_6_]^2−^, and as a result, it was much lower than that of [SiF_6_]^2−^ after a few days. The [SiF_6_]^2−^ drove the crystal growth, which promoted the growth of KSF on the surface of KSFM crystals, forming a surface with low Mn^4+^ content. As shown in Figure 6b, the KSFM single crystal has a cubic shape and six chamfers. As the growth time was prolonged from 6 h to 4 days, the average size of KSFM crystals increased from 200 µm to 1 mm. Zhong et al. [50] used a similar method to add a certain molar mass of Na_2_SiF_6_: Li^+^(LNSF) powder to the mixed solution of KMnO_4_, KF·2H_2_O, and HF and then stirred it for 48 h at room temperature. With the passing of time, the [MnF_6_]^2−^ on the crystal surface gradually decreased, forming a shell almost free of Mn^4+^. LNSF: Mn^4+^ crystals were obtained by washing. The water resistance of single crystals was positively correlated with crystallization time. In particular, Jiang et al. [61] introduced K_2_SiF_6_: Mn^4+^ to saturate the HF solution of K_2_SiF_6_ and stirred. After stirring for a duration of time, K_2_SiF_6_: Mn^4+^ @ K_2_SiF_6_ could be synthesized by adding the appropriate amount of ethanol to cause the epitaxial growth of the K_2_SiF_6_: Mn^4+^ shell and then synthesized K_2_SiF_6_: Mn^4+^ @ K_2_SiF_6_. The addition of ethanol could also enhance the emission intensity of K_2_SiF_6_: Mn^4+^. When the concentration of Mn^4+^ was 4% mol, the PL intensity of the product with the addition of ethanol was 5.03 times higher than that without the addition of ethanol.

The saturated solution crystallization method can construct the core–shell structure at one time, which reduces the experimental operation steps, and yields phosphor with fewer surface defects. Compared with the other two methods, it has unique advantages and provides a new idea for constructing the core–shell structure. However, if the crystallization process is subjected to external forces, the influence on the experimental results is particularly obvious. When the saturated solution is impacted or stirred, the quality and size of the crystal will be affected. This is caused by the balance between nucleation and growth during crystallization, which may even degrade the optical properties. Moreover, this method requires more time to ensure the formation of the core–shell structure.

## 4. Luminescent Mechanism of A_2_MF_6_: Mn^4+^

**Figure 7:**
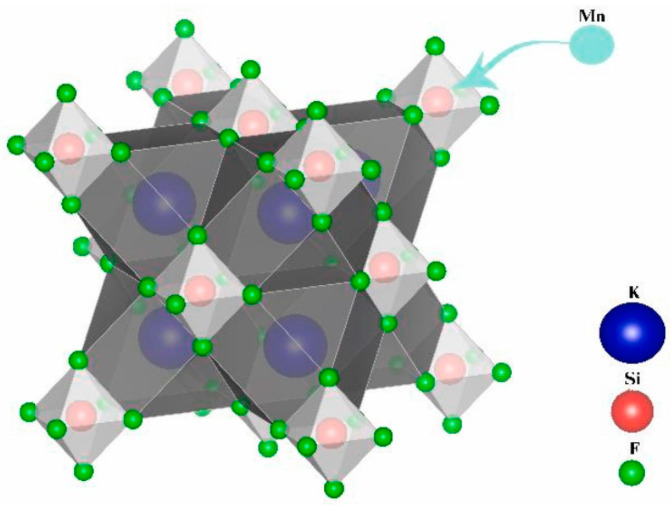
The crystal structure of K_2_SiF_6_: Mn^4+^. (Reprinted from Ref [65]. Copyright from Elsevier, 2022).

In A_2_MF_6_: Mn^4+^, each M^4+^ ion is surrounded by six F^−^ ions, forming a [MF_6_]^2−^octahedral structure. Furthermore, the A^+^ ions are located in the center of 12 adjacent F^−^ ions, forming a regular polyhedron. Figure 7 shows the schematic crystal structure of K_2_SiF_6_: Mn^4+^. The overall and local structure of A_2_MF_6_: Mn^4+^ are similar. There is also a clear overlap in excitation and emission spectra. Mn^4+^ has two broad excitations at 300–500 nm and a series of narrow-band emissions in the 600–650 nm range. The optical properties of some transition metal ions with ^3^d_3_ structures (such as Mn^4+^, Cr^3+^, and V^2+^) can all be explained by the Tanabe–Sugano energy diagram [64]. The electronic energy levels of ^3^d_3_ transition metal ions are affected by D_q_/B, where D_q_ is the crystal field strength, and B is the Racah parameter. The energies of most multiparticle are strongly dependent on the crystal field strength, except for the ^2^T_1g_ and ^2^E_g_ energy levels parallel to the ground state ^4^A_2g_. The strength of D_q_ depends on the distance between the ions and the ligand R. High energy states such as ^4^T_2g_, ^4^T_1g_, and ^2^A_1g_ can be modulated by R and the type of ligand.

According to the d–d transition rule, the ^4^A_2g_→^4^T_1g_ and ^4^A_2g_→^4^T_2g_ of Mn^4+^ are spin-allowed transitions, which are located in the near ultraviolet (UV) region of 300–400 nm and the blue region of 400–500 nm, respectively. When they are excited to the ^4^T_1g_ or ^4^T_2g_ level, the excited ions usually relax non-radiatively to ^2^E_g_, and the ^2^E_g_→^4^A_2g_ transition emission is inhibited by both spin and equivalence. However, due to the coupling between electrons and phonons, the emission is partially unlocked, and there are many sharp narrow band emission peaks in the range of 610–650 nm. The optical transition of Mn^4+^ ions is sensitive to its local coordination. In a symmetric low-host crystal, Mn^4+^ ions can partially break the odd–even forbidden transition rule, resulting in a strong zero phonon line (ZPL), which is generally located at about 620 nm. The emission peaks on the left and right sides of ZPL are the anti-Stokes peak and the Stokes peak, respectively. Strong ZPL can improve the red-light purity of Mn^4+^-doped red phosphors, which is beneficial for WLED applications.

## 5. Fluorescent Properties and Water Resistance

It was found that A_2_MF_6_: Mn^4+^ was very sensitive to humidity, and the Mn^4+^ dopant on the surface was easy to hydrolyze into mixed manganese oxides and hydroxides, which darkened the color and extremely weakened its red emission intensity. For moisture-sensitive materials, coating a waterproof layer on the surface was an effective method to raise its stability. Mn^4+^-doped fluoride was unstable in the water environment, and its water resistance was improved by constructing a core–shell structure. Generally, fluoride is exposed to high temperature (80 °C, HT) and high humidity (80%, HH), water, or boiling water for a period of time, and the effect of water resistance is measured by the remaining photoluminescence (PL) intensity. In addition, the water resistance could also be measured by the contact angle between the phosphor and water. Moreover, compared with A_2_MF_6_: Mn^4+^ without the shell, some of the A_2_MF_6_: Mn^4+^ with the core–shell structure not only improved the water resistance but also had a certain degree of improvement in fluorescence performance and thermal stability.

### 5.1. A_2_MF_6_: Mn^4+^ with Heterogeneous Shell Layer

**Figure 8:**
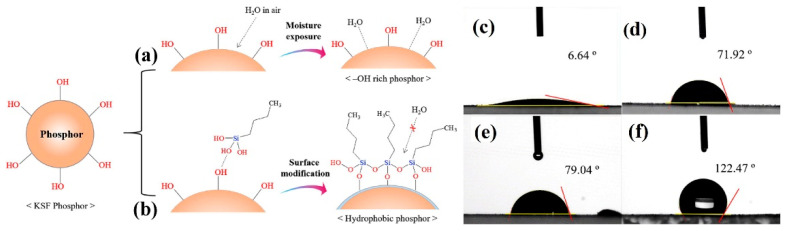
The formation mechanism of (**a**) adsorption of water and (**b**) moisture-proof layer on K_2_SiF_6_: Mn^4+^ surface; water contact angle image of (**c**) KSF; (**d**) C3-KSF; (**e**) C6-KSF; (**f**) C16-KSF. (Reprinted from Ref [37]. Copyright from Elsevier, 2017).

**Figure 9:**
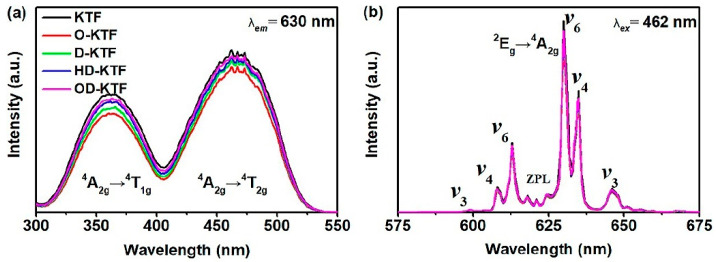
(**a**) Excitation and (**b**) emission spectra of K_2_TiF_6_ after surface modification. (Reprinted from Ref [36]. Copyright from Elsevier, 2017).

Nguyen et al. [35] coated K_2_SiF_6_: Mn^4+^ (KSFM-MOPAl) with an alkyl phosphate layer. The yellow KSFM phosphor turned brown after immersion in water for 30 min, while KSFM-MOPAl remained yellow after immersion in water for 60 min. The non-radiative transition activation energy of the coated KSFM-MOPAl phosphor (0.92 eV) was 0.09 eV higher than that of the uncoated KSFM phosphor (0.83 eV). As a result, the temperature of thermal quenching was improved, and the comprehensive PL intensity of KSFM-MOPAl at 523K was 100% of that at room temperature. However, with the increase of shell thickness, the luminescence intensity of phosphor decreased slightly. Kim et al. [37] modified K_2_SiF_6_: Mn^4+^(KSFM) with an alkyl coupling agent, as shown in Figure 8a,b. The surface of the unmodified phosphor had hydrophilic -OH groups, which combined with water molecules in the air, leading to structure destruction and optical property degradation of the phosphor. Silane particles reacted with -OH bunches on the surface of the fluoride to create a thin layer on its surface, which effectively protected the phosphor from external moisture. In Figure 8c,f, the initialization value of the water contact angle was 6.64°, while after surface modification, the angle expanded to 122.47° after surface modification. Superhydrophobic silane coupling agents (SCAs) were employed by Zhou et al. [36] to modify coating surfaces and increase the moisture resistance of Mn^4+^-activated fluoride phosphors. As shown in Figure 9, the fluorescence intensity of K_2_TiF_6_ almost did not weaken after surface modification. After surface modification with OTMS, DTMS, HDTMS, and ODTMS solutions, the water contact angle of K_2_TiF_6_ significantly increased from nearly 0° to 36.8°, 52.7°, 147.9°, and 155.4°, respectively. Compared with the experimental results of Kim et al. [37], both of them formed hydrophobic organic layers on the surface of phosphors, which significantly reduced the -OH or H_2_O molecules on the surface of the phosphors and improved the water-resistance of the phosphor, and the hydrophobicity increased as the carbon chain of silane became longer. However, the experiment of Zhou et al. had higher feasibility because it was simple to operate and was carried out at room temperature, and it also caused the phosphor to have better moisture resistance. Arunkumar et al. [38] used oleic acid (OA) as a hydrophobic layer, and the hydrophobic tail of OA formed a thin layer to protect phosphors from atmospheric moisture and prevent the hydrolysis of [MnF_6_]^2−^. K_2_SiF_6_-OA and DenKa-based K_2_SiF_6_ phosphors were aged at HT and HH for 450 h and then packaged into WLEDs. The emission intensity of K_2_SiF_6_-OA-WLED decreased by 15%, while the commercial DenKa-based K_2_SiF_6_-WLED decreased by 23% under the device current of 120 mA. The water stability and efficient red emission of the encapsulated K_2_SiF_6_@OA phosphors indicated that employing OA to treat fluorine phosphors was a feasible method and could be apply in WLED. However, the synthesis temperature of this method was high, and there was a risk of toxic by-products. Luo et al. [40] used pyruvate to prepare KFSM-98PA with a bilayer shell, and its internal quantum efficiency (IQE) value was as high as 99.71%. However, due to the influence of the shell, the PL intensity was 90% of the original KSFM. After soaking in water for 360 h, the PL intensity was maintained at 88.5% of the original intensity, while the shell-free KSFM was only 51.6%. Moreover, the color of KSFM-98PA did not change, even after boiling in water for 1 h. In addition, PA could recover the fluorescence of hydrolyzed fluoride, and the IQE of hydrolyzed h-KSFM could recover up to 95.24%.

**Figure 10:**
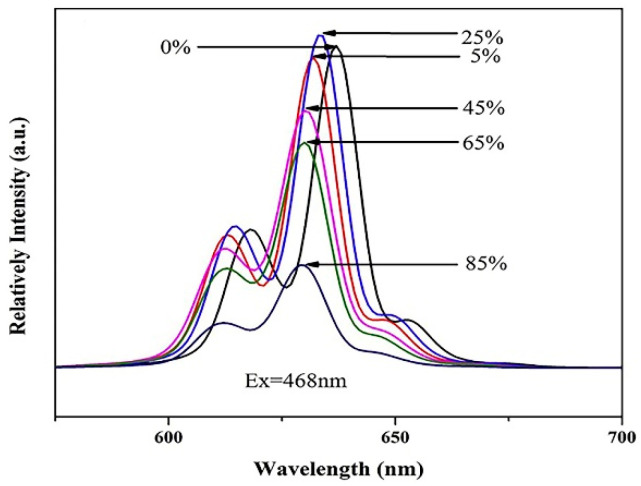
Emission spectra of K_2_TiF_6_: Mn^4+^@CaF_2_. (Reprinted from Ref [41]. Copyright from Elsevier, 2019).

Compared with K_2_TiF_6_, the luminescence intensity of the K_2_TiF_6_@CaF_2_ phosphor prepared by Dong et al. [41] increased slightly. The change of luminescence intensity after surface modification was mainly due to the removal of surface hydroxyl groups by surface coating modification, which enhanced the emission. As shown in Figure 10, with the change of the CaF_2_ coating amount, the KTF@CaF_2_ had a blue shift of 2–5 nm. The CaF_2_ coating changed the crystal field environment around Mn^4+^, resulting in a blue shift in its luminescence. After 2 h of immersion, the luminescence intensity of KTF@CaF_2_ decreased by only 13.6%, while KTF decreased by 93.2%. Yu et al. [63] prepared K_2_SiF_6_: Mn^4+^@CaF_2_ with high water resistance and thermal stability by the hydrothermal method and surface coating process. Compared with KSF: Mn^4+^, the CaF_2_ shell could effectively prevent the hydrolysis of surface [MnF_6_]^2−^ groups to MnO_2_. After soaking in water for 6 h, the luminescence intensity of the uncoated product decreased to 41.68% of the initial product, while the coated material was 88.24% of the initial value. Compared with Dong’s KTF: Mn^4+^@CaF_2_, KSF: Mn^4+^@CaF_2_ had a negative thermal quenching effect (NTQ), and the mechanism can be considered to be a thermal-light energy conversion mechanism. Fang et al. [43] modified the SrF_2_ shell on the K_2_TiF_6_:Mn^4+^ surface, but the PL intensity slightly decreased with the amount of SrF_2_ due to increased light scattering and absorption at the core–shell interface. The PL intensity of K_2_TiF_6_: Mn^4+^@SrF_2_ phosphor soaked in distilled water for 2 h maintained more than 90% of the initial value, while the PL of K_2_TiF_6_: Mn^4+^ was only 21.2% of the initial value. The results showed that the SrF_2_ shell could effectively cut off the hydrolysis of the inner [MnF_6_]^2−^ groups. Quan et al. [46] synthesized K_2_SiF_6_@SiO_2_, which had good thermal stability in the range of 300 °C. The PL intensity was 100% of the initial value at 250 °C and 75% at 300 °C, which was better than the thermal stability of K_2_SiF_6_ (82% at 250 °C and 27% at 300 °C). After soaking in water for 1 h, the luminescence intensity values of K_2_SiF_6_@SiO_2_ and K_2_SiF_6_ were 43% and 7% of the initial values, respectively. SiO_2_ could significantly improve the water-resistance of K_2_SiF_6_, but K_2_SiF_6_@SiO_2_ would hydrolyze in a short time, and its external quantum efficiency was as low as 0.494%.

**Figure 11:**
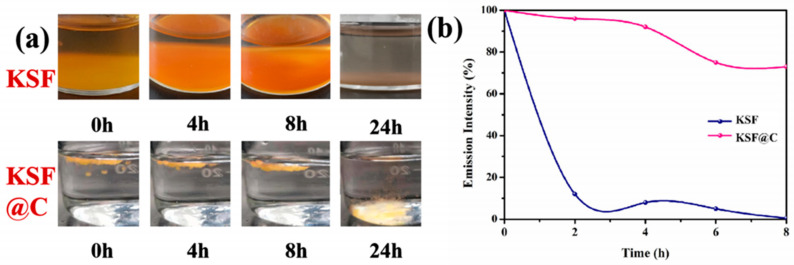
(**a**) The photographs of K_2_SiF_6_: Mn^4+^ and K_2_SiF_6_: Mn^4+^@C after immersion for different times; (**b**) emission intensity curves of K_2_SiF_6_: Mn^4+^ and K_2_SiF_6_: Mn^4+^@C phosphors after hydrolysis for different times. (Reprinted from Ref [48]. Copyright from Elsevier, 2020).

Yu et al. [47] synthesized (i) K_2_SiF_6_: Mn^4+^, (ii) K_2_SiF_6_: Mn^4+^, Na^+^, (iii) K_2_SiF_6_: Mn^4+^, Na^+^@GQDS, and (iv) K_2_SiF_6_: Mn^4+^, Na^+^@GQDs@K_2_SiF_6_ by the hydrothermal method and the room temperature coating method. The PL intensity values of (ii), (iii), and (iv) were 1.21, 1.47, and 1.71 times that of (i), respectively, which means that Na^+^ co-doping, GQDs, and KSF shells could further enhance the emission intensity of the samples. At 180 °C, (iii) and (iv) had obvious NTQ effects because their PL intensity values at 180 °C were 217% and 298% of that at 30 °C, respectively. The mechanism of NTQ was further regarded as thermo-optic energy conversion. After being immersed in deionized water for 360 min, the emission intensities of (iii) and (iv) decreased from 100% to 70.57% and 91.63%, respectively. The water resistance of KSFM with a double coating structure improved significantly. As shown in Figure 11, the K_2_SiF_6_: Mn^4+^@C phosphor synthesized by Liu et al. [48] could maintain 73% of the initial luminescence intensity after soaking in aqueous solution for 8 h at room temperature, while K_2_SiF_6_: Mn^4+^ was only 0.7% of the initial value under the same conditions. Although the C deposition layer could improve the water-resistance of KSF, it also decreased the luminescence intensity of KSF.

Among the coating materials, alkyl coupling agents, oleic acid, pyruvate, CaF_2_, and nano-carbon can improve the water resistance of the material. Alkyl phosphate, SiO_2_, and GQDs have certain effects on the enhancement of the water resistance of the materials, but they are not ideal enough. Among them, GQD shells can also improve the thermal stability of Mn^4+^-doped fluoride, which possess the obvious NTQ effect. The heterogeneous shell isolates the luminescent center [MnF_6_]^2−^ from the external moisture, which decreases the water sensitivity of the Mn^4+^-doped fluoride. However, some areas of phosphor particles cannot be coated entirely due to the uneven and incomplete deposition of the coating material.

### 5.2. A_2_MF_6_: Mn^4+^ with Homogeneous Shell Layer

**Figure 12:**
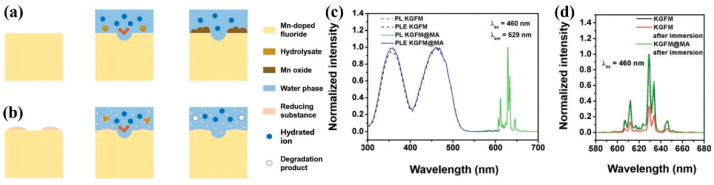
(**a**) Schematic diagram of water corrosion of K_2_GeF_6_: Mn^4+^ and (**b**) K_2_GeF_6_: Mn^4+^@MA; (**c**) normalized excitation emission spectra for K_2_GeF_6_: Mn^4+^ and K_2_GeF_6_: Mn^4+^@MA; (**d**) normalized emission spectra of K_2_GeF_6_: Mn^4+^ and K_2_GeF_6_: Mn^4+^@MA before and after immersion in water for 168 h. (Reprinted from Ref [49]. Copyright from Royal Society of Chemistry, 2018).

**Figure 13:**
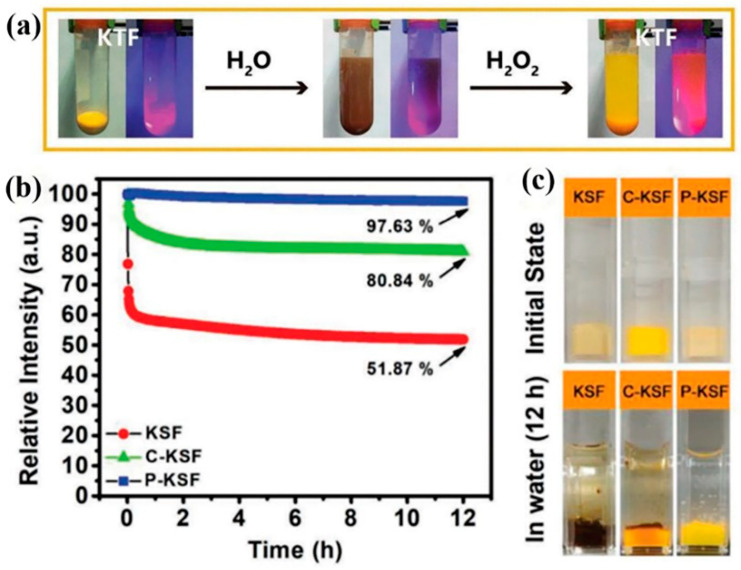
(**a**) Photos of KTF phosphors repaired with H_2_O_2_; (**b**) the relative PL intensity of KSF, P-KSF, and C-KSF soaked in water for different times; (**c**) comparison of KSF, P-KSF, and C-KSF before and after soaking in water for 12 h. (Reprinted from Ref [51]. Copyright from John Wiley and Sons Ltd., 2018).

Huang et al. [49] decomposed the oxides and hydroxide of Mn^4+^ in situ in an aqueous environment, as shown in Figure 12a,b. The aqueous solution produced by the hydrolysis of KGFM was brown. The aqueous solution of KGFM improved in transparency and clearness after the addition of DL-mandelic acid (MA), and the optical properties of KGFM could also be recovered. Figure 12c,d shows that KGFM@MA had the same high luminescence performance as KGFM. After 168 h of immersion in water, the luminescence intensity of KGFM@MA was almost unchanged (98%), while the luminescence intensity of KGFM was only 33% of the original. As a mild reducing acid material, MA could decompose brown hydrolases in situ to recover the optical properties of fluoride. Furthermore, it was also suitable for other commercial materials to improve the water-resistance, such as K_2_SiF_6_: Mn^4+^ and K_2_TiF_6_: Mn^4+^, which provided a new approach for synthesizing water-resistant Mn^4+^-doped fluoride phosphors with narrow bands. After 168 h of a water erosion experiment, the PL intensities of the original KSFM and KTFM remained at 38.7% and 12.8%, respectively, which was significantly lower than 100% of KSFM@MA and 108% of KTFM@MA. Huang et al. [50] synthesized red phosphors by coating and surface passivation methods, respectively. After soaking in water for 6 h, the WR-KSFM-8 synthesized by the surface passivation method retained 76% of its initial emission intensity, which was much higher than 11% that of IE-KSFM synthesized by the coating method. Zhou et al. [51] reestablished the luminescence properties of hydrolyzed phosphor by adding H_2_O_2_. As shown in Figure 13a, the hydrolysis of Mn^4+^ quickly reacted with H_2_O_2_ until the surface oxidation–reduction was complete and the internal [MnF_6_]^2−^ groups were separated from the external moisture. As shown in Figure 13b,c, the same humidity test was performed on P-KSF and the commercial water-resistance phosphor powder C-KSF. Their wet strength values were 97.63% and 80.84% of their initial values, respectively. The passivation surface of the H_2_O_2_ aqueous solution provided an effective method for constructing core–shell structures, and the fluoride phosphor prepared by this method had high water-resistance and did not need to consume additional HF solution. The surface redox method could be extended to other doping systems, opening up a new perspective for the development of luminescent materials that enhance the stability of the surface and the duration of the device. Liu et al. [52] synthesized CSFM-P by passivating Cs_2_SiF_6_: Mn^4+^ with H_2_O_2_ by low-temperature coprecipitation. Compared with the original phosphor CSFM, the emission intensity of the original phosphor decreased to 13.6% after immersion in water for 168 h, while CSFM-P still maintained 74.0% of the initial value. Under excitation at 460 nm, the IQE, EQE, and absorption efficiency of CSFM-P phosphor was 98%, 85%, and 86.8%, whereas the corresponding values of the commercial KSFM were 92%, 67.08%, and 72.92%, respectively.

**Figure 14:**
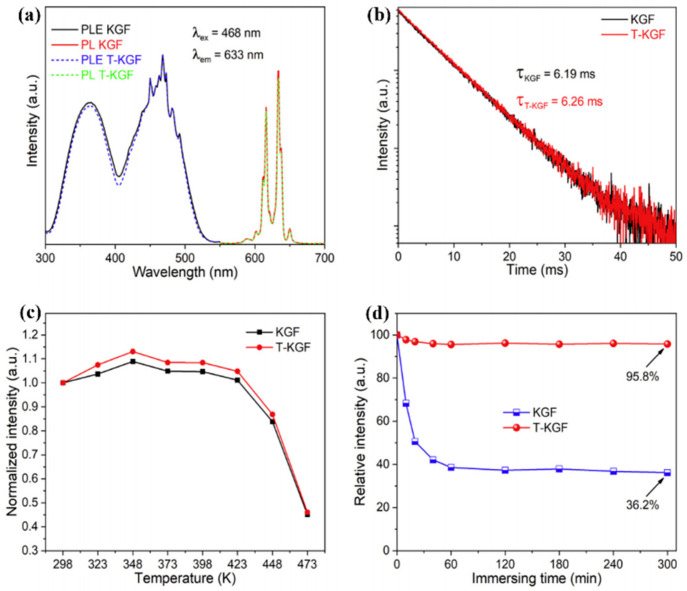
(**a**) Excitation and emission spectra of KGF and T-KGF; (**b**) decay curves KGF and T-KGF; (**c**) temperature-dependent relative PL intensities KGF and T-KGF; (**d**) relative PL intensities of immersion in water for different times of KGF and T-KGF. (Reprinted from Ref [53]. Copyright from Elsevier, 2018).

**Figure 15:**
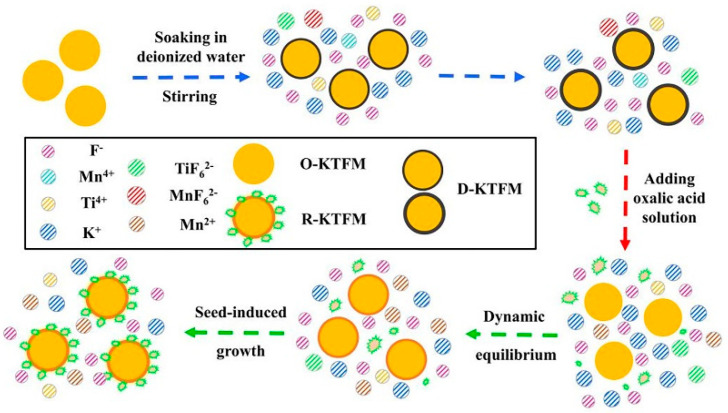
Schematic illustration of the reverse strategy of K_2_SiF_6_: Mn^4+^ to restore luminescence and improve moisture-resistance. (Reprinted from Ref [55]. Copyright from John Wiley and Sons Ltd., 2020).

**Figure 16:**
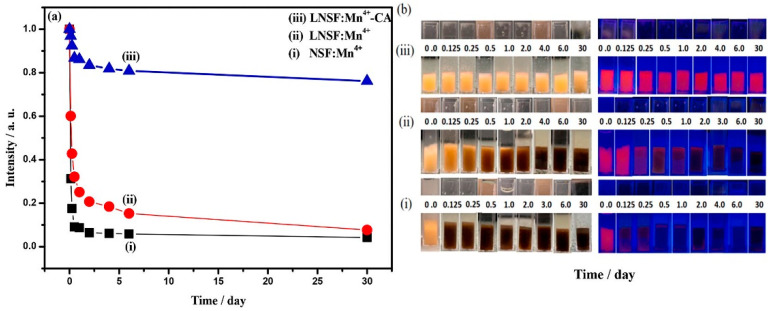
(**a**) Comprehensive PL intensity; (**b**) photographs in natural light and under 365 nm UV light. (i) NSF: Mn^4+^, (ii) LNSF: Mn^4+^, and (iii) water resistance of LNSF: Mn^4+^-CA soaked in deionized water for 30 days. (Reprinted from Ref [57]. Copyright from Elsevier, 2022).

Yu et al. [53] constructed K_2_GeF_6_: Mn^4+^@K_2_GEF_6_ (T-KGF) with a uniform Mn^4+^-free surface layer. As shown in Figure 14a, the position and shape of the characteristic peaks did not change significantly. H_2_C_2_O_4_ solution treatment did not affect the luminescence characteristics of Mn^4+^ ions in the K_2_GeF_6_ host, because the PL intensity, fluorescence lifetime, and thermal quenching behavior were nearly unchanged. Figure 14c,d show that the emission intensity of T-KGF kept 95.8% of the initial value while that of KGF was only 36.2% of the original value after 5 h of immersion in water. The disadvantage was that the internal Mn^4+^ ions were difficult to reduce. Jiang et al. [54] passivated Rb_2_SnF_6_: Mn^4+^ with oxalic acid to construct a protective layer of Mn^4+^ ion passivation on the surface. The PL intensity of Rb_2_SnF_6_: Mn^4+^ decreased to a certain extent. After soaking in boiling water for 3 h, the PL intensity of shell Rb_2_SnF_6_: Mn^4+^ was 95% of that at room temperature (RT) after soaking in boiling water for 3 h. Liu et al. [55] successfully restored the luminescence characteristics of hydrolyzed K_2_SiF_6_: Mn^4+^ with oxalic acid and significantly improved the water resistance. Figure 15 showed a schematic diagram of the reverse strategy to restore luminescence and enhance water resistance. During the repair process, oxalic acid reacted with hydrolyzed dark brown material to reduce Mn^4+^ ions to soluble low-state Mn ions, releasing K, Si, and F elements into the supernatant. The solubility of K_2_SiF_6_ in solution was particularly low, and it would precipitate out and form a K_2_SiF_6_ shell on the surface of K_2_SiF_6_: Mn^4+^ particles. The emission intensity of K_2_SiF_6_: Mn^4+^ phosphors repaired by oxalic could reestablish to 103.68% of the original K_2_SiF_6_: Mn^4+^ red phosphors (O-KSFM). The recouped K_2_SiF_6_: Mn^4+^ (R-KSFM), kept around 62.3% of the beginning relative emission, escalated after 5 h of submersion in deionized water. Moreover, the luminescence intensity of the degraded K_2_TiF_6_: Mn^4+^ (D-KTFM) fluorophores could recover 162.59% of the original K_2_TiF_6_: Mn^4+^ (O-KTFM). Zhong et al. [57] used surface passivation to synthesize LiNaSiF_6_: Mn^4+^-CA, Li^+^ co-doping, and surface passivation improved the luminescence intensity of LiNaSiF_6_: Mn^4+^. As shown in Figure 16, after 3 and 6 h of immersion, the PL intensity of NSF: Mn^4+^ decreased to 31.26 and 17.54%, LNSF: Mn^4+^ decreased to 60.11% and 42.73%, and LNSF: Mn^4+^-CA maintained 96.84% and 92.33% of the initial value, respectively. Even after immersion in water for 30 days, the luminescence intensity of LNSF: Mn^4+^-CA was still 76.16% of the original. LNSF: Mn^4+^-CA had 154% and 118% fluorescence intensities at 120 °C and 150 °C compared with that at 30 °C, respectively. The NTQ effect could be explained by the large number of electron traps formed by LNSF: Mn^4+^-CA after surface passivation. As the temperature increased, the electrons obtained additional energy compensation from the electron traps generated by the matrix defects and then transferred the energy to Mn^4+^ ions, inducing the NTQ effect. The luminescence intensity of CSNage_0.5_Sn_0.5_F_6_: Mn^4+^ synthesized by Li et al. [56] decreased sharply after immersion in water for more than 5 min, which may be caused by the fact that the matrix CNGSF with double-central ions is more easily hydrolyzed due to larger crystal distortion. The luminescence intensity of the phosphors C1-CNGSFM and C2-CNGSFM modified by citric acid or oxalic acid slightly increased in the first 2 h, which may have been due to the diffusion of the phosphor in water. The reflection and refraction of the excitation light were increased, while the PL intensity remained unchanged for 16 h. When the quenched CNGSFM was stirred in the modifier for 30 min, the luminescence of CNGSFM could be restored to the initial brightness. Cai et al. [58] treated K_2_SiF_6_ with FeCl_2_ to form a protective shell on the surface and obtained T-KSF, whose PL intensity was significantly higher than KSF, effectively reducing the non-radiative transition probability of Mn^4+^ ions. The Mn^4+^-free shell protected the T-KSF particles and effectively reduced the luminescence center ions in the particles that were in direct contact. After immersion for 320 min, the relative luminescence intensity of the KSF phosphors decreased sharply to only 63.4% of the initial intensity value, while the T-KSF samples still maintained 80.3% of the initial intensity.

**Figure 17:**
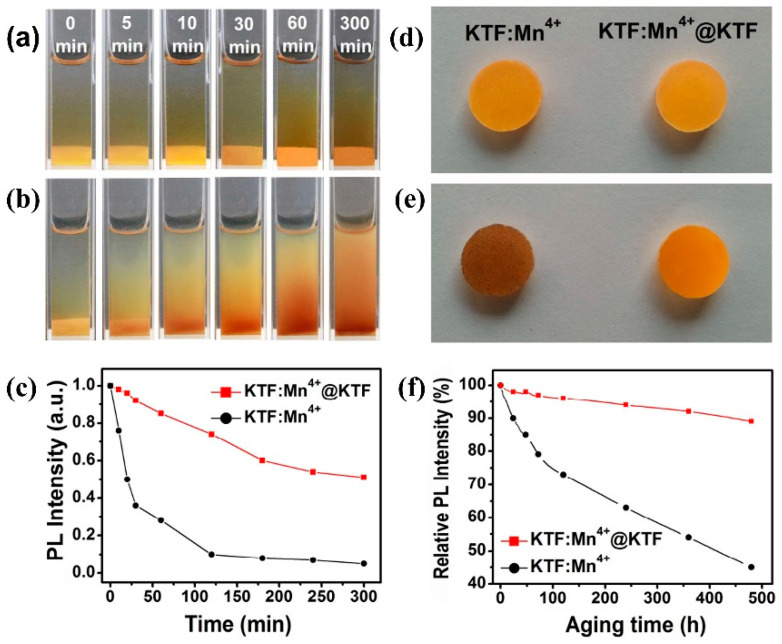
(**a**) Photographs of KTF: Mn^4+^@KTF and (**b**) KTF: Mn^4+^ phosphors soaked for different times; (**c**) comprehensive PL intensities of KTF: Mn^4+^@KTF and KTF: Mn^4+^ soaked for different times; (**d**) before and (**e**) after the phosphors aged under HT and HH conditions photographs; (**f**) comprehensive PL intensity of the phosphors aging at different times. (Reprinted from Ref [28]. Copyright from John Wiley and Sons Ltd., 2019.).

The K_2_SiF_6_: Mn^4+^@K_2_SiF_6_ synthesized by Li et al. [59] kept the initial PL intensity of 88% after soaking in water for 300 min, while the strength of the uncoated sample decreased to 1%. Moreover, the PL intensities of K_2_SiF_6_: Mn^4+^@K_2_SiF_6_ at 120 °C, 150 °C, 180 °C, and 210 °C were 176%, 198%, 214%, and 213% of the initial PL intensities at 30 °C, respectively. The K_2_SiF_6_ coating had multiple effects on the luminescence properties of K_2_SiF_6_: Mn^4+^ red phosphors. In addition to preventing the hydrolysis of Mn^4+^, the energy transfer to surface defects was also prevented. The probability of radiative transition increased with temperature more quickly than that of non-radiative transition. Huang et al. [28] synthesized K_2_TiF_6_: Mn^4+^@K_2_TiF_6_ phosphors by the reverse ion strategy. As shown in Figure 17a,b, the yellow KTF: Mn^4+^ sample quickly changed to brown after 5 min, while the yellow KTF: Mn^4+^@KTF remained yellow even after 5 h in water. The relative fluorescence intensity can be seen in Figure 17c. The relative PL intensities of KTF: Mn^4+^@KTF and KTF: Mn^4+^ after aging 480 h in the HT and HH environment are shown in Figure 17f, which retained 89% and 45% of the initial value, respectively.

**Figure 18:**
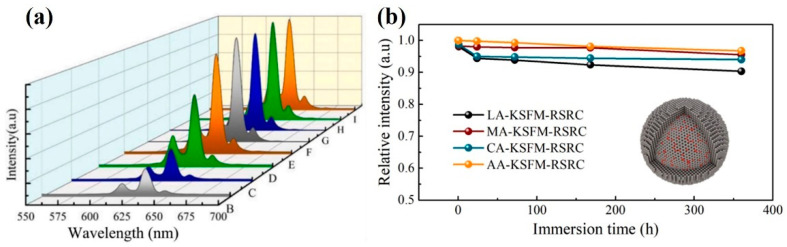
(**a**) PL spectra of KSFM (F, G, H, I) and after boiling in water (B, C, D, E) for 20 min, (B, F) KSFM, (C, G) KSFM-CE, (D, H) KSFM-SP, (E, I) KSFM-RSRC; (**b**) Relative PL intensities of KSFM-RSRC treated with LA, MA, CA and AA immersed in water for different times. (Reprinted from Ref [29]. Copyright from Elsevier, 2021).

**Figure 19:**
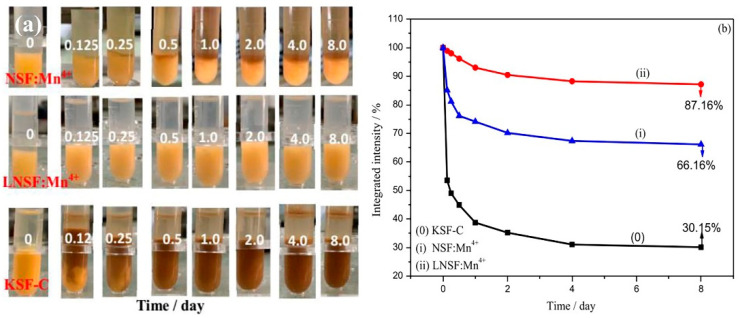
Na_2_SiF_6_: Mn^4+^, LiNaSiF_6_: Mn^4+^, K_2_SiF_6_: Mn^4+^-C soaked in deionized water for different times: (**a**) photographs under natural light; (**b**) relative PL changes. (Reprinted from Ref [60]. Copyright from American Chemical Society, 2022).

Wan et al. [29] proposed a new idea of reduction-assisted surface recrystallization (RSRC) to reconstruct the Mn^4+^-free shell of fluoride. Using α-hydroxy acid in the RSRC process could improve the water-resistance of the fluoride. The PL intensity of KSFM-RSRC fluoride containing LA, MA, CA, and AA was maintained at 90%, 96%, 94%, and 97% of the initial PL intensity after the soaking for 360 h in water, respectively, as shown in Figure 18a. They also prepared KSFM-SP and KSFM-CE phosphors by surface passivation and cation exchange, respectively. The PL intensities of KSFM phosphors treated with RSRC, SP, and CE maintained 97%, 100%, and 97% of the original KSFM, respectively. The PL intensity of KSFM did not decrease by different treatments. As presented in Figure 18b, after boiling in water for 20 min, the PL intensities of KFSM-RSRC, KFSM-SP, KFSM-CE, and the original KSFM were maintained at 96%, 65%, 31%, and 25% of that before boiling, respectively. In contrast, the core–shell fluoride constructed by the reduction-assisted surface recrystallization method had better water resistance. Compared with the commercial K_2_SiF_6_: Mn^4+^-CP, K_2_SiF_6_: Mn^4+^ prepared by Zhou et al. [30] had better thermal stability and water-resistance. There was almost no quenching at 200 °C, the fluorescence intensity was 100.8% of the initial value, which was almost unchanged, while the luminescence intensity of KFSM-CP was just 87.1%. After soaking in deionized water for 12 h, the initial values were 97.6% and 80.8%, respectively. KSFM single crystal fluorophores formed an almost Mn^4+^-free surface and possessed excellent water resistance. LiNaSiF_6_: Mn^4+^ with a core–shell structure prepared by Zhong et al. [50] had an NTQ effect, while the Na_2_SiF_6_: Mn^4+^ did not. The PL intensity values at 150 °C were 148% and 43% of those at 30 °C, respectively. The thermal stability of Mn^4+^ was even greater than that of K_2_SiF_6_: Mn^4+^-C (commercial K_2_SiF_6_: Mn^4+^). As shown in Figure 19, after soaking in water, the fluorescence intensities of the LiNaSiF_6_: Mn^4+^, Na_2_SiF_6_: Mn^4+^, and K_2_SiF_6_: Mn^4+^-C samples maintained 87.16%, 66.16%, and 30.15% of their initial values, respectively. Co-doping Li^+^ not only led to carrier transfer (CT), which induced the NTQ effect, but it also led to the surface formation, the CT produced the NTQ effect, and the surface prevented the hydrolysis of Mn^4+^ on the surface of the sample. Jiang et al. [61] used anti-solvent to induce the epitaxial growth of deposited fluoride to prepare K_2_SiF_6_: Mn^4+^@K_2_SiF_6_, which retained 82% of the initial emission intensity after submerging in water for 4 h and retained 90% of the initial emission intensity after 10 days under HT and HH conditions, while K_2_SiF_6_: Mn^4+^ only retained 38% after immersion for 4 h. The epitaxial development of the deposition caused by ethanol improved the water-resistance and luminescence of K_2_SiF_6_: Mn^4+^.

The core–shell structure of K_2_SiF_6_: Mn^4+^ coated with an alkyl phosphate layer improved the water resistance of the phosphor. Subsequently, a series of Mn^4+^-doped fluorides based on a core–shell structure was successfully synthesized. Table 1 shows the comparison of water resistance and thermal stability of A_2_MF_6_: Mn^4+^ fluoride with and without the core–shell structure reported recently. It is clear from the table that the relative PL intensity of A_2_MF_6_: Mn^4+^ fluoride with the core–shell structure was higher than that of Mn^4+^-doped fluorides without the shell, whether in the HT and HH condition or soaking in water for a long time, which showed that the water-resistance was significantly improved. Moreover, the thermal stability of some A_2_MF_6_: Mn^4+^ fluorides was also improved. Thus, the core–shell structure could markedly increase the water-resistance and stability of A_2_MF_6_: Mn^4+^ fluorides. As summarized above, the homogeneous shell layer included an organic shell, such as alkyl phosphates, octadecyl trimethoxy silanes, silane coupling agents, oleic acid, pyruvate, and the inorganic shell, such as SiO_2_, Al_2_O_3_, TiO_2_, CaF_2_, nano-carbon, GQD, SrF_2_, and the homogeneous shell layer coated by the same material as itself. These shell materials can improve the water resistance of A_2_MF_6_: Mn^4+^, but the luminescence intensities of A_2_MF_6_: Mn^4+^ may be affected by the transparency and thickness of the shell layer material, especially the heterogeneous shell layer. The homogeneous shell is constructed by the coating construction method, which is the earliest method to construct the core–shell structure of A_2_MF_6_: Mn^4+^. The synthetic process of this method is mature. However, there are certain requirements for the synthesis conditions and equipment, which may involve high temperature and high-pressure conditions. At the same time, the existence of a shell interface may affect the optical properties of the phosphors, resulting in the shell falling off [44]. Therefore, when using this method, the shell layer material that is more matched with the lattice of the matrix can be selected to reduce the influence of the interface. The homogeneous shell layer is mainly constructed by the surface passivation method and the saturated crystallization method. The synthesis process of the surface passivation method is simple. Homogeneous shell layer phosphors can be formed by stirring with the reducing agent solution, such as DL-mandelic acid, oxalic acid, citric acid, L-tartaric acid, DL-malic acid, ascorbic acid, DL-lactic acid, H_3_PO_4_/H_2_O_2_, and FeCl_2_, for a period of time. This method requires the matrix itself to be slightly soluble or insoluble in water to obtain excellent water resistance. The saturated crystallization method can synthesize single crystals with high crystallinity and few defects. However, this method also requires the solubility of the matrix material. In addition, the saturated crystallization method needs more time to ensure the formation of a shell free of Mn^4+^ ions. The homogeneous core–shell structure crystallized with the saturated solution has better water-resistance, which may be due to the presence of fewer Mn^4+^ ions on the shell surface, and the core is coated completely, while the surface passivation method may leave more Mn^4+^ ions on the surface due to an insufficient redox reaction. It is hoped that these summaries will be helpful for the design, synthesis, and optical performance optimization of A_2_MF_6_: Mn^4+^ with improved water resistance in the future.

## 6. Application of A_2_MF_6_: Mn^4+^ in WLED

A_2_MF_6_: Mn^4+^ exhibits a series of narrow sharp emissions within the wavelength range of 600–650 nm, showing unique excitation and emission characteristics, making it a candidate for WLED, and especially K_2_SiF_6_: Mn^4+^ has been commercialized. Huang et al. [50] packaged ternary (WR-K_2_SiF_6_: Mn^4+^-8) and binary (without red phosphor) WLEDs using blue LED chips. The correlation color temperature (CCT), color rendering index (Ra), and the luminous efficacy (LE) of binary WLED were 5661 K, and 69.8 and 168 lm/W, respectively, while the CCT, Ra, and LE values of ternary WLED were 5398 K, and 80.5 and 96 lm/W, respectively. Obviously, with the addition of A_2_MF_6_: Mn^4+^ red phosphor, ternary WLED did have lower CCT and higher color rendering index than binary WLED. However, the LE of ternary WLED greatly decreased. Therefore, more attention should be paid to the trade-offs between Ra, R9, CCT, and LE of WLED according to the field of practical application. Table 2 lists the basic optoelectronic parameters of recently reported WLED devices based on A_2_MF_6_: Mn^4+^ red phosphors with or without the core–shell structure. As listed in it, the color rendering index (such as Ra and R9) and LE of A_2_MF_6_: Mn^4+^ were not distinctive, regardless of whether it possessed the shell layer, indicating that the core–shell structure had little effect on the luminescent properties of the WLED. However, it had a significant effect on the service life of the device. Zhou et al. [51] packaged WLEDs by using YAG&K_2_TiF_6_: Mn^4+^ (LED-1), and YAG&P-K_2_TiF_6_: Mn^4+^ (LED-2). When LED-1 was aged in HT and HH conditions for 60 days, its LE began to decrease, and it finally failed after aging for about 77 days. The LE of LED-2 also gradually decreased during the aging process, but it still remained at 39.5% of the initial LE after being aged in HT and HH conditions for 100 days. Obviously, the A_2_MF_6_: Mn^4+^ deteriorates much less than the shell-free fluoride under HT and HH conditions. Thus, the improved water resistance of A_2_MF_6_: Mn^4+^ by constructing a core–shell can significantly prolong the lifetime of WLED devices, which is beneficial to environmental protection and resource saving.

## 7. Conclusions

The market demand for WLEDs of high quality is still growing rapidly. Compared with rare earth doped red phosphors, A_2_MF_6_: Mn^4+^ red phosphors have strong broadband excitation at nearly 460 nm and stable narrow-band red emission at about 630 nm, as well as the advantages of high thermal stability, low cost, and room temperature synthesis, which are beneficial to the development of WLED. Many research results summarized in this review show that constructing core–shell structures is an effective approach to improve the moisture resistance of A_2_MF_6_: Mn^4+^ red phosphors and avoid the degradation of device performance caused by the deliquescence of the phosphors. There are still some challenges to be further studied to meet the commercial application requirements. Firstly, high concentrations of HF can decompose KMnO_4_ to produce manganese (VI), provide F^−^ ions in an aqueous solution to obtain [MnF_6_]^2−^ complexes instead of MnO_2_, and facilitate the incorporation of Mn^4+^ into the host lattice, thereby enhancing the luminescent efficiency of phosphors. However, as a corrosive and volatile acid, HF introduces hazards in terms of safety and the environment. Although low toxicity H_3_PO_4_/KHF_2_ have been used to replace highly toxic HF and good luminescent efficiency has been achieved, Mn^4+^ is prone to be reduced to Mn^2+^ under high temperature and high humidity conditions, which decreases the luminescent properties. Hence, it is urgent to develop new green synthesis routes with low HF or without HF without sacrificing the luminescent performance and water resistance. Secondly, there is no uniform standard for measuring the water resistance of A_2_MF_6_: Mn^4+^ red phosphors. Different dosages and different test methods always show different results of water resistance. Therefore, relevant test standards should be developed, which is very important in terms of commercial applications. Thirdly, some optoelectronic parameters of WLED can be improved by adding red powder. However, some other parameters (such as the luminous efficacy) may be decreased at the same time. Thus, from the device perspective, more attention needs to be paid to the trade-offs between Ra, R9, CCT, and light efficiency, as well as the costs and lifespans of WLEDs. Moreover, though many Mn^4+^-doped fluoride red phosphors have been discovered in the past few decades, it is still valuable to explore new Mn^4+^-doped fluoride red phosphors with more excellent comprehensive performances to meet the demand of commercialization.

## Figures and Tables

**Table 1:** Comparison of water resistance and thermal stability of A_2_MF_6_: Mn^4+^ red phosphor with and without core–shell structures.

Phosphor	Shell	Water Resistance				Thermal Stability		Ref.
		HT HH Storage Time/h	PL Intensity Relative to Room Temperature/%	Soaking Time in Water/h	PL Intensity Relative to Room Temperature/%	Temperature/°C	PL Intensity Relative to Room Temperature/%	
K_2_SiF_6_: Mn^4+^@OA K_2_SiF_6_: Mn^4+^	Oleic acid None	450	85% 77%	-	-	-	-	[38]
K_2_SiF_6_: Mn^4+^-98PA K_2_SiF_6_: Mn^4+^	Pyruvate None	360	88.5% 51.6%	-	-	-	-	[40]
K_2_SiF_6_: Mn^4+^@CaF_2_ K_2_SiF_6_: Mn^4+^	CaF_2_ None	-	-	6	88.24 41.68	210	2.07 1.93	[63]
K_2_SiF_6_: Mn^4+^@SiO_2_ K_2_SiF_6_: Mn^4+^	SiO_2_ None	-	-	1	43 7	250	100 82	[46]
K_2_SiF_6_:Mn^4+^, Na^+^@GQDs@K_2_SiF_6_ K_2_SiF_6_: Mn^4+^, Na^+^@GQDs	GQDs@K_2_SiF_6_ GQDs	-	-	6	91.63 70.57	180	298 217	[47]
K_2_SiF_6_: Mn^4+^@C K_2_SiF_6_: Mn^4+^	C None	-	-	8	73 0.7	-	-	[48]
WR-K_2_SiF_6_: Mn^4+^-8 IE-K_2_SiF_6_: Mn^4+^	K_2_SiF_6_ K_2_SiF_6_	-	-	6	76 11	-	-	[50]
R-K_2_SiF_6_: Mn^4+^ K_2_SiF_6_: Mn^4+^	K_2_SiF_6_ None	-	-	5	62.3	150	111.9 106.7	[55]
LA-K_2_SiF_6_: Mn^4+^-RSRC MA-K_2_SiF_6_: Mn^4+^-RSRC CA-K_2_SiF_6_: Mn^4+^-RSRC AA-K_2_SiF_6_: Mn^4+^-RSRC	K_2_SiF_6_ K_2_SiF_6_ K_2_SiF_6_ K_2_SiF_6_	-	-	360	90 96 94 97	-	-	[29]
T-K_2_SiF_6_: Mn^4+^ K_2_SiF_6_: Mn^4+^	K_2_SiF_6_ None	-	-	5.3	80.3 63.4	-	-	[58]
K_2_SiF_6_: Mn^4+^@K_2_SiF_6_ K_2_SiF_6_: Mn^4+^	K_2_SiF_6_ None	-	-	5	88 1	120	213	[59]
K_2_SiF_6_: Mn^4+^-CP K_2_SiF_6_: Mn^4+^	K_2_SiF_6_ None	-	-	12	97.6 80.8	200	100.8 87.1	[30]
K_2_SiF_6_: Mn^4+^@K_2_SiF_6_ K_2_SiF_6_: Mn^4+^	K_2_SiF_6_ None	240	90 -	4	82 38	-	-	[61]
LiNaSiF_6_: Mn^4+^-CA LiNaSiF_6_: Mn^4+^	LiNaSiF_6_ None	-	-	6	92.33 42.73	150	118	[57]
LiNaSiF_6_: Mn^4+^ Na2SiF6: Mn4+	LiNaSiF_6_	-	-	182	87.16 66.16	-	-	
Cs_2_SiF_6_: Mn^4+^-P Cs_2_SiF_6_: Mn^4+^	Cs_2_SiF_6_ None	-	-	168	74 13.6	152	101	[52]
K_2_TiF_6_: Mn^4+^@CaF_2_ K_2_TiF_6_: Mn^4+^	CaF_2_ None	-	-	2	86.4 6.8	-	-	[41]
K_2_TiF_6_: Mn^4+^@SrF_2_ K_2_TiF_6_: Mn^4+^	SrF_2_ None	-	-	2	80.3 63.4	-	-	[43]
P-K_2_TiF_6_: Mn^4+^ C-K_2_TiF_6_: Mn^4+^ K_2_TiF_6_: Mn^4+^	K_2_TiF_6_ K_2_TiF_6_ None	-	-	12	97.63 80.84 51.87	-	-	[50]
K_2_TiF_6_: Mn^4+^@K_2_TiF_6_	K_2_TiF_6_	480	89	-	-	-	-	[28]
K_2_GeF_6_: Mn^4+^@MA K_2_GeF_6_: Mn^4+^	K_2_GeF_6_ None	-	-	168	98 33	-	-	[49]
T-K_2_GeF_6_:Mn^4+^ K_2_GeF_6_:Mn^4+^	K_2_GeF_6_ None	-	-	5	95.8 36.2	-	-	[53]
Rb_2_SnF_6_:Mn^4+^	Rb_2_SnF_6_	-	-	3 (Boiling water)	95	-	-	[54]

**Table 2:** Basic optoelectronic parameters of WLED devices encapsulated with A_2_MF_6_: Mn^4+^ red phosphor.

Phosphor	Shell	Current/mA	Ra	R9	CCT/K	LE	Ref
K_2_SiF_6_: Mn^4+^@CaF_2_	CaF_2_	20	89.3		3956	-	[63]
K_2_SiF_6_: Mn^4+,^ Na^+^@GQDs@ K_2_SiF_6_	GQDs@ K_2_SiF_6_	20	91.3	-	4546	-	[47]
K_2_SiF_6_: Mn^4+^@K_2_SiF_6_	K_2_SiF_6_	20	80.5	63.8	5398	96	[50]
R-K_2_SiF_6_: Mn^4+^	K_2_SiF_6_	20	90.4	94.2	2892	183.31	[55]
T-K_2_SiF_6_: Mn^4+^	K_2_SiF_6_	20	-	-	3500	81.6	[58]
K_2_SiF_6_: Mn^4+^@K_2_SiF_6_	K_2_SiF_6_	20	91.3	-	3326	100.5	[59]
R-K_2_SiF_6_: Mn^4+^	K_2_SiF_6_	20	90.4	94.2	2892	183.31	[55]
K_2_SiF_6_: Mn^4+^@K_2_SiF_6_	K_2_SiF_6_	20	91.3	-	3326	100.5	[59]
K_2_SiF_6_: Mn^4+^@K_2_SiF_6_	K_2_SiF_6_	20	-	-	2929	119.74	[61]
LiNaSiF_6_: Mn^4+^-CA LNSF: Mn^4+^	LiNaSiF_6_ None	20	89.6 74.6	-	3916 3939	109.6 107.8	[57]
LiNaSiF_6_: Mn^4+^	LiNaSiF_6_	20	90.4	89	3173	122	[60]
CsSiF_6_: Mn^4+^-P	CsSiF_6_	20	-	-	6880	133	[52]
K_2_GeF_6_: Mn^4+^@K_2_GeF_6_	K_2_GeF_6_	20	86.3	-	3824	152.37	[53]

## Data Availability

Not applicable.

## References

[1] Li G.G., Tian Y., Zhao Y., Lin J. (2015). Recent progress in luminescence tuning of Ce^3+^ and Eu^2+^-activated phosphors for pc-WLEDs. Chem. Soc. Rev..

[2] Li J.H., Zhang Z.H., Li X.H., Xu Y.Q., Ai Y.Y., Yan J., Shi J.X., Wu M.M. (2017). Luminescence properties and energy transfer of YGa_1.5_Al_1.5_(BO_3_)_4_: Tb^3+^, Eu^3+^ as a multi-colour emitting phosphor for WLEDs. J. Mater. Chem. C.

[3] Chen Y.J., Xing W.S., Liu Y.X., Zhang X.S., Xie Y.Y., Shen C.Y., Liu J.G.X., Geng C., Xu S. (2020). Efficient and Stable CdSe/CdS/ZnS Quantum Rods-in-Matrix Assembly for White LED Application. Nanomaterials.

[4] Oh J.H., Eo Y.J., Yoon H.C., Huh Y.-D., Do Y.R. (2016). Evaluation of new color metrics: Guidelines for developing narrow-band red phosphors for WLEDs. J. Mater. Chem. C.

[5] Wang L., Wang X., Kohsei T., Yoshimura K., Izumi M., Hirosaki N., Xie R.J. (2015). Highly efficient narrow-band green and red phosphors enabling wider color-gamut LED backlight for more brilliant displays. Opt. Express.

[6] Feng X.Y., Jiang K., Zeng H.B., Lin H.W. (2019). A Facile Approach to Solid-State White Emissive Carbon Dots and Their Application in UV-Excitable and Single-Component-Based White LEDs. Nanomaterials.

[7] Zhou Z., Zhou N., Xia M., Yokoyama M., Hintzen H.T. (2016). Research progress and application prospects of transition metal Mn^4+^-activated luminescent materials. J. Mater. Chem. C.

[8] Zhu H., Lin C.C., Luo W., Shu S., Liu Z., Liu Y., Kong J., Ma E., Cao Y., Liu R.S. (2014). Highly efficient non-rare-earth red emitting phosphor for warm white light-emitting diodes. Nat. Commun..

[9] Liang S.S., Shang M.M., Lian H.Z., Li K., Zhang Y., Lin J. (2017). An efficient rare-earth free deep red emitting phosphor for improving the color rendering of white light-emitting diodes. J. Mater. Chem. C.

[10] Lin C.C., Liu R.S. (2011). Advances in Phosphors for Light-emitting Diodes. J. Phys. Chem. Lett..

[11] Liu X.F., Qiu J.R. (2015). Recent advances in energy transfer in bulk and nanoscale luminescent materials: From spectroscopy to appli cations. Chem. Soc. Rev..

[12] Lu W., Lv W.Z., Zhao Q., Jiao M.M., Shao B.Q., You H.P. (2014). A novel efficient Mn^4+^ activated Ca_14_Al_10_Zn_6_O_35_ phosphor: Applica tion in red-emitting and white LEDs. Inorg. Chem..

[13] Blum O., Shaked N.T. (2015). Prediction of photothermal phase signatures from arbitrary plasmonic nanoparticles and experimental verification. Light Sci. Appl..

[14] Vanetsev A., Põdder P., Oja M., Khaidukov N.M., Makhov V.N., Nagirnyi V., Romet I., Vielhauer S., Mändar H. (2021). Microwave-hydrothermal synthesis and investigation of Mn-doped K_2_SiF_6_ microsize powder as a red phosphor for warm white LEDs. J. Lumin..

[15] Du J., Poelman D. (2019). Facile Synthesis of Mn^4+^-Activated Double Perovskite Germanate Phosphors with Near-Infrared Persistent Luminescence. Nanomaterials.

[16] Hong F., Yang L., Xu H.P., Chen Z., Liu Q.X., Liu G.X., Dong X.G., Yu W.S. (2019). A red-emitting Mn^4+^ activated phosphor with controlled morphology and two-dimensional luminescence nanofiber film: Synthesis and application for high-performance warm white light-emitting diodes (WLEDs). J. Alloys Compd..

[17] Adachi S. (2018). Photoluminescence properties of Mn^4+^-activated oxide phosphors for use in white-LED applications: A review. J. Lumin..

[18] Adachi S. (2018). Photoluminescence spectra and modeling analyses of Mn^4+^-activated fluoride phosphors: A review. J. Lumin..

[19] Lang T.C., Han T., Fang S.Q., Wang J.Y., Cao S.X., Peng L.L., Liu B.T., Korepanov V.I., Yakovlev A.N. (2020). Improved phase stability of the metastable K_2_GeF_6_:Mn^4+^ phosphors with high thermal stability and water-proof property by cation substitution. Chem. Eng. J..

[20] Hong F., Cheng H., Song C., Liu G.X., Yu W.S., Wang J., Dong X.T. (2019). Novel polygonal structure Mn^4+^ activated In^3+^-based Elpasolite-type hexafluorides red phosphor for warm white light-emitting diodes (WLEDs). Dalton Trans..

[21] Adachi S., Takahashi T. (2008). Direct synthesis and properties of K_2_SiF_6_:Mn^4+^ phosphor by wet chemical etching of Si wafer. J. Appl. Phys..

[22] Jansen T., Baur F., Jüstel T. (2017). Red emitting K_2_NbF_7_: Mn^4+^ and K_2_TaF_7_: Mn^4+^ for warm-white LED applications. J. Lumin..

[23] Jin Y., Fang M.H., Grinberg M., Mahlik S., Lesniewski T., Brik M.G., Luo G.Y., Lin J.G., Liu R.S. (2016). Narrow Red Emission Band Fluoride Phosphor KNaSiF_6_:Mn^4+^ for Warm White Light-Emitting Diodes. ACS Appl. Mater. Interfaces.

[24] Liao J.S., Nie L.L., Zhong L.F., Gu Q.J., Wang Q. (2016). Co-precipitation synthesis and luminescence properties of K_2_TiF_6_:Mn^4+^ red phosphors for warm white light-emitting diodes. Luminescence.

[25] Tang F., Su Z.C., Ye H.G., Wang M.Z., Lan X., Phillips D.L., Cao Y.G., Xu S.J. (2016). A set of manganese ion activated fluoride phosphors (A_2_BF_6_:Mn^4+^, A = K, Na, B = Si, Ge, Ti): Synthesis below 0 °C and efficient room-temperature photoluminescence. J. Mater. Chem. C.

[26] Zhu M.M., Pan Y.X., Xi L.Q., Lian H.Z., Lin J. (2017). Design, preparation, and optimized luminescence of a dodec-fluoride phosphor Li_3_Na_3_A_l2_F_12_:Mn^4+^ for warm WLED applications. J. Mater. Chem. C..

[27] Zhu Y.W., Cao L.Y., Brik M.G., Zhang X.J., Huang L., Xuan T.T., Wang J. (2017). Facile synthesis, morphology and photolumines cence of a novel red fluoride nanophosphor K_2_NaAlF_6_:Mn^4+^. J. Mater. Chem. C.

[28] Huang D.C., Zhu H.M., Deng Z.H., Zou Q.L., Lu H.Y., Yi X.D., Guo W., Lu C.D., Chen X.Y. (2019). Moisture-Resistant Mn^4+^-Doped Core-Shell-Structured Fluoride Red Phosphor Exhibiting High Luminous Efficacy for Warm White Light-Emitting Diodes. Angew. Chem. Int. Ed..

[29] Wan P.P., Liang Z.J., Luo P.L., Lian S.X., Zhou W.L., Liu R.-S. (2021). Reconstruction of Mn^4+^-free shell achieving highly stable red-emitting fluoride phosphors for light-emitting diodes. Chem. Eng. J..

[30] Zhou Y.Y., Yu C.K., Song E.H., Wang Y.J., Ming H., Xia Z.G., Zhang Q.Y. (2020). Three Birds with One Stone: K_2_SiF_6_:Mn^4+^ Single Crystal Phosphors for High-Power and Laser-Driven Lighting. Adv. Opt. Mater..

[31] Noh M., Yoon D.H., Kim C.H., Lee S.J. (2019). Organic solvent-assisted synthesis of the K_3_SiF_7_:Mn^4+^ red phosphor with improved morphology and stability. J. Mater. Chem. C.

[32] Adachi S. (2022). Review-Negative Thermal Quenching of Mn^4+^ Luminescence in Fluoride Phosphors: Effects of the 4A2g → 4T2g Excitation Transitions and Normal Thermal Quenching. ECS J. Solid State Sci. Technol..

[33] Kim Y.H., Ha J., Im W.B. (2021). Towards green synthesis of Mn^4+^-doped fluoride phosphors: A review. J. Mater. Res. Technol..

[34] Yan S. (2020). Critical Review—On the Anomalous Thermal Quenching of Mn^4+^ Luminescence in A_2_XF_6_:Mn^4+^ (A = K, Na, Rb or Cs; X = Si, Ti, Ge, Sn, Zr or Hf). ECS J. Solid State Sci. Technol..

[35] Nguyen H.D., Lin C.C., Liu R.S. (2015). Waterproof Alkyl Phosphate Coated Fluoride Phosphors for Optoelectronic Materials. Angew. Chem..

[36] Zhou Y.Y., Song E.H., Deng T.T., Zhang Q.Y. (2018). Waterproof Narrow-Band Fluoride Red Phosphor K_2_TiF_6_:Mn^4+^ via Facile Superhydrophobic Surface Modification. ACS Appl. Mater. Interfaces.

[37] Kim J., Jang I., Song G.Y., Kim W.-H., Jeon S.-W., Kim J.-P. (2018). Controlling surface property of K_2_SiF_6_:Mn^4+^ for improvement of lighting-emitting diode reliability. J. Phys. Chem. Solids.

[38] Arunkumar P., Kim Y.H., Kim H.J., Unithrattil S., Im W.B. (2017). Hydrophobic Organic Skin as a Protective Shield for Moisture-Sensitive Phosphor-Based Optoelectronic Devices. ACS Appl. Mater. Interfaces.

[39] Fang M.H., Hsu C.S., Su C., Liu W., Wang Y.H., Liu R.S. (2018). Integrated Surface Modification to Enhance the Luminescence Properties of K_2_TiF_6_:Mn^4+^ Phosphor and Its Application in White-Light-Emitting Diodes. ACS Appl. Mater. Interfaces.

[40] Luo P.L., Ye M.L., Zhou W.L., Wan P.P., Ma Z.Y., Qiu Z.X., Zhang J.L., Liu R.-S., Lian S.X. (2022). Simultaneous construction of impermeable dual-shell stabilizing fluoride phosphors for white light-emitting diodes. Chem. Eng. J..

[41] Dong Q.Z., Guo C.J., He L., Lu X.F., Yin J.B. (2019). Improving the moisture resistance and luminescent properties of K_2_TiF_6_:Mn^4+^ by coating with CaF_2_. Mater. Res. Bull..

[42] Li Y.L., Zhong X., Yu Y., Liu Y.M., Liao S., Huang Y.H., Zhang H.X. (2021). H_2_O_2_-free preparation of K_2_SiF_6_:Mn^4+^ and remarkable high luminescent thermal stability induced by coating with graphene quantum dots. Mater. Chem. Phys..

[43] Fang Z.Y., Lai X.H., Zhang J., Zhang R. (2021). Surface modification of K_2_TiF_6_:Mn^4+^ phosphor with SrF_2_ coating to enhance water resistance. Int. J. Appl. Ceram. Technol..

[44] Verstraete R., Rampelberg G., Rijckaert H., Van Driessche I., Coetsee E., Duvenhage M.-M., Smet P.F., Detavernier C., Swart H., Poelman D. (2019). Stabilizing Fluoride Phosphors: Surface Modification by Atomic Layer Deposition. Chem. Mater..

[45] Ten Kate O.M., Zhao Y.J., Jansen K.M.B., Ruud van Ommen J., Hintzen H.T. (2019). Effects of Surface Modification on Optical Properties and Thermal Stability of K_2_SiF_6_:Mn^4+^ Red Phosphors by Deposition of an Ultrathin Al_2_O_3_ Layer Using Gas-Phase Deposition in a Fluidized Bed Reactor. ECS J. Solid State Sci. Technol..

[46] Quan V.T.H., Tuyet D.T., Dereń P.J., Hieu N.P.T., Duy N.H. (2019). Feasible preparation of red-phosphor K_2_SiF_6_:Mn^4+^ coated with SiO_2_ for white light emitting diodes application. Vietnam J. Chem..

[47] Yu Y., Wang T.M., Deng D.S., Zhong X., Li Y.L., Wang L., Liao S., Huang Y.H., Long J.Q. (2022). Enhancement of the luminescent thermal stability and water resistance of K_2_SiF_6_: Mn^4+^, Na^+^ by double coating of GQDs and K_2_SiF_6_. J. Alloys Compd..

[48] Liu Y.X., Hu J.X., Ju L.C., Cai C., Hao V.B., Zhang S.H., Zhang Z.W., Xu X., Jian X., Yin L.J. (2020). Hydrophobic surface modification toward highly stable K_2_SiF_6_:Mn^4+^ phosphor for white light-emitting diodes. Ceram. Int..

[49] Huang L., Liu Y., Si S.C., Brik M.G., Wang C.X., Wang J. (2018). A new reductive dl-mandelic acid loading approach for moisture-stable Mn^4+^ doped fluorides. Chem. Commun..

[50] Huang L., Liu Y., Yu J.B., Zhu Y.W., Pan F.J., Xuan T.T., Brik M.G., Wang C.X., Wang J. (2018). Highly Stable K_2_SiF_6_:Mn^4+^@K_2_SiF_6_ Composite Phosphor with Narrow Red Emission for White LEDs. ACS Appl. Mater. Interfaces.

[51] Zhou Y.Y., Song E.H., Deng T.T., Wang Y.J., Xia Z.G., Zhang Q.Y. (2019). Surface Passivation toward Highly Stable Mn^4+^-Activated Red-Emitting Fluoride Phosphors and Enhanced Photostability for White LEDs. Adv. Mater. Interfaces.

[52] Liu Y., Zhou Z., Huang L., Brik M.G., Si S.C., Lin L.T., Xuan T.T., Liang H.B., Qiu J.B., Wang J. (2019). High-performance and moisture-resistant red-emitting Cs_2_SiF_6_:Mn^4+^ for high-brightness LED backlighting. J. Mater. Chem. C.

[53] Yu H.J., Wang B.C., Bu X.Y., Liu Y.-g., Chen J., Huang Z.H., Fang M.H. (2020). A facile in situ surface-coating passivation strategy for improving the moisture resistance of Mn^4+^-activated fluoride red phosphor. Ceram. Int..

[54] Jiang C.Y., Brik M.G., Srivastava A.M., Li L.H., Peng M.Y. (2019). Significantly conquering moisture-induced luminescence quench ing of red line-emitting phosphor Rb_2_SnF_6_:Mn^4+^ through H_2_C_2_O_4_ triggered particle surface reduction for blue converted warm white light-emitting diodes. J. Mater. Chem. C.

[55] Liu L., Wu D., He S.G., Ouyang Z., Zhang J.F., Du F., Peng J.Q., Yang F.L., Ye X.L. (2020). A Reverse Strategy to Restore the Mois ture-deteriorated Luminescence Properties and Improve the Humidity Resistance of Mn^4+^-doped Fluoride Phosphors. Chem. Asian. J..

[56] Li D., Pan Y.X., Wei X.N., Lin J. (2021). Significantly enhanced the humidity resistance of a novel red phosphor CsNaGe_0.5_Sn_0.5_F_6_:Mn^4+^ through surface modification. Chem. Eng. J..

[57] Zhong X., Deng D.S., Wang T.M., Li Y.L., Yu Y., Qiang J.W., Liao S., Huang Y.H., Long J.Q. (2022). A facile surface passivation strategy for Na_2_SiF_6_:Mn^4+^, Li^+^ phosphors to achieve high moisture resistance and luminescent thermal stability. J. Lumin..

[58] Cai W.T. (2021). [RETRACTED]: Highly Stable Mn^4+^-Activated Red-Emitting Fluoride Phosphors and Enhanced moisture stability for White LEDs. E3S Web Conf..

[59] Li Y.L., Yu Y., Zhong X., Liu Y.M., Chen L., Liao S., Huang Y.H., Zhang H.X. (2021). K_2_SiF_6_:Mn^4+^@K_2_SiF_6_ phosphor with remarkable negative thermal quenching and high water resistance for warm white LEDs. J. Lumin..

[60] Zhong X., Deng D.S., Wang T.M., Li Y.L., Yu Y., Qiang J.W., Liao S., Huang Y.H., Long J.Q. (2022). High Water Resistance and Luminescent Thermal Stability of Li_y_Na_2-y_SiF_6_:Mn^4+^ Red-Emitting Phosphor Induced by Codoping of Li. Inorg. Chem..

[61] Jiang C.Y., Li L.H., Brik M.G., Lin L.T., Peng M.Y. (2019). Epitaxial growth via anti-solvent-induced deposition towards a highly efficient and stable Mn^4+^ doped fluoride red phosphor for application in warm WLEDs. J. Mater. Chem. C.

[62] Wang Z.-L., Guo R., Li G.-R., Ding L.-X., Ou Y.-N., Tong Y.-X. (2011). Controllable synthesis of ZnO-based core/shell nanorods and core/shell nanotubes. RSC Adv..

[63] Yu Y., Wang T.M., Zhong X., Li Y.L., Wang L., Liao S., Huang Y.H., Long J.Q. (2021). High luminescent thermal stability and water resistance of K_2_SiF_6_:Mn^4+^@CaF_2_ red emitting phosphor. Ceram. Int..

[64] Xu H.P., Hong F., Pang G., Liu G.X., Dong X.T., Wang J.X., Yu W.S. (2020). Co-precipitation synthesis, luminescent properties and application in warm WLEDs of Na_3_GaF_6_:Mn^4+^ red phosphor. J. Lumin..

[65] Subhoni M., Zafari U., Srivastava A.M., Beers W.W., Cohen W., Brik M.G., Yamamoto T. (2021). First-principles investigations of geometrical and electronic structures of Mn^4+^ doped A_2_SiF_6_ (A = K, Rb, Cs) red phosphors. Opt. Mater..

